# Novel Camptothecin Derivative 9c with Enhanced Antitumor Activity via NSA2-EGFR-P53 Signaling Pathway

**DOI:** 10.3390/ijms26051987

**Published:** 2025-02-25

**Authors:** Fu Du, Aotong Zhang, Xin Qi, Ruijuan Yin, Tao Jiang, Jing Li

**Affiliations:** 1 Key Laboratory of Marine Drugs, Chinese Ministry of Education, School of Medicine and Pharmacy, Ocean University of China, Qingdao 266003, China; dufu@stu.ouc.edu.cn (F.D.); zhangaotong6069@stu.ouc.edu.cn (A.Z.); qixin_ouc@ouc.edu.cn (X.Q.); yrj928@163.com (R.Y.); 2 Laboratory for Marine Drugs and Bioproducts of Qingdao National, Laboratory for Marine Science and Technology, Qingdao 266003, China

**Keywords:** camptothecin derivatives, NSA2, P53, EGFR, antitumor

## Abstract

Therapeutic challenges persist in the management of non-small cell lung cancer (NSCLC) in oncology. Camptothecins have demonstrated as crucial agents in tumor therapy; however, their efficacy is significantly hindered by adverse effects and drug resistance. Herein, we present a novel camptothecin derivative named **9c**, which exhibits impressive anti-NSCLC potency surpassing the widely recognized camptothecin analog **FL118** through a novel mechanism. Our findings demonstrated that **9c** effectively inhibited tumor malignancy through cell cycle arrest and apoptosis induction with the transcriptional downregulation of anti-apoptotic genes including *survivin*, *Mcl-1*, *Bcl-2*, and *XIAP*. Mechanistically, **9c** induced a wild-type p53 expression by destabilizing the NSA2-EGFR axis, thus delaying the cell cycle progression and ultimately triggering apoptosis. **9c** significantly inhibited the growth of the NSCLC xenograft in vivo without observed side toxicity. Importantly, it complemented the therapeutic advantages of the novel drug AMG510 for addressing KRAS-mutant NSCLC. Collectively, these findings position **9c** as a promising candidate with innovative approaches to combat NSCLC.

## 1. Introduction

Lung cancer is one of the most common malignant tumors in the world, of which non-small cell lung cancer (NSCLC) is the majority, accounting for more than 80% of lung cancer [1]. The current therapeutic modalities for NSCLC include surgical resection, radiation therapy, chemotherapy, targeted therapies, and immunotherapy [2]. Chemotherapy is still the primary treatment for NSCLC, with cisplatin, docetaxel, gemcitabine (GEM), irinotecan, paclitaxel, and vinorelbine as the mainstay therapeutic agents [3]. However, although these drugs produce a good initial response and slightly increase the overall survival time of patients, patients will still inevitably develop drug resistance, resulting in poor treatment effects. In recent years, the use of immunotherapy in NSCLC holds great promise, but the clinical application of the approaches has been limited due to their low response and resistance. The emergence of precision medicine has heralded a new era in the treatment of non-small cell lung cancer (NSCLC), significantly expanding therapeutic options for patients with advanced stages of the disease. Although a variety of molecular targets are being explored for NSCLCs, such as EGFR, ALK, MEK and KRAS, etc. [4,5], which offer a means for the development of drugs to combat tumors through genetic and epigenetic cues, effective treatments for lung cancer without known specific targeting molecules remain elusive. Furthermore, it seems that the development of mutation-tailored drugs may be approaching a plateau, as recent exome sequencing studies of non-small cell lung cancer (NSCLC) have not uncovered a significant number of novel, potentially druggable targets; thus, NSCLC therapy remains an unsolved challenge in oncology. Notably, many newly developed compounds are focused on exceptionally rare genetic events recently [6,7,8], which paves an avenue to expedite the development of novel anti-cancer agents.

**Figure 1:**
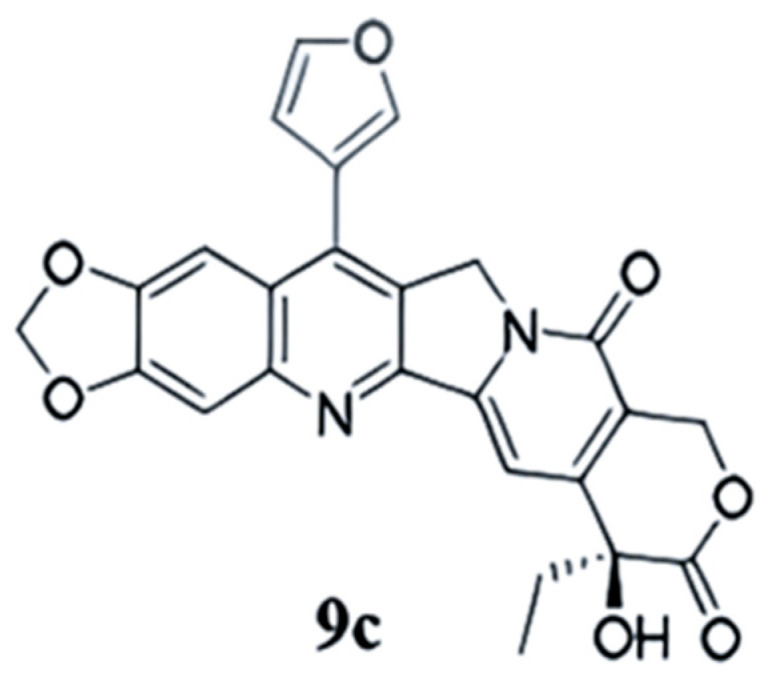
Chemical structure of **9c**.

The quinoline alkaloid camptothecin (CPT), as a topoisomerase I (Topo I) inhibitor, is a famous isolated pharmaceutical agent [9]. In the past decades, a plethora of CPT derivatives has been synthesized and evaluated. Among these, semi-synthetic analogs like topotecan and irinotecan have garnered approval from the U.S. Food and Drug Administration (FDA) for the treatment of various malignancies, including lung cancer [10,11,12]. **FL118**, the (20S)-10,11-methylenedioxy-camptothecin, is a camptothecin analog similar in structure to irinotecan and exhibits remarkable anti-tumor efficacy across a spectrum of cancer types in vitro and in vivo [13,14]. Studies have revealed that, despite sharing an identical core structure with its counterparts, the antitumor efficacy of **FL118** remains robust and unimpaired in the absence of targeted Topo I. Instead, **FL118** inhibits gene promoter activities and endogenous expressions of anti-apoptotic proteins, such as survivin, Mcl-1, XIAP, and cIAP2 in a selective manner [13,15], thereby promoting to cell apoptosis induction and antitumor activity. Moreover, **FL118** exhibits its remarkable efficacy independent of p53 status (wild-type, mutant, or null) in cells, and, thus, it stands as a potent therapeutic agent for cancers characterized by p53 dysfunction, in which most DNA damage drugs (if not all) show a remarkable lack of efficiency [13,16]. Nevertheless, the poor water solubility and adverse reactions of **FL118** have seriously hindered its further research [15,17]. Revisiting the structure–activity relationship of camptothecin, as illuminated by the existing literature, reveals that 7-lipophilic substitution significantly amplifies its cytotoxicity and anti-tumor efficacy, even with the introduction of large lipophilic substituents and long-chain substituents, which may be related to rapid intracellular accumulation and lactone stability [18,19,20]. Evidence supports that several 7-substituted camptothecins have the ability to overcome drug resistance both in vitro and in vivo [21,22,23]. Furthermore, the strategic introduction of an appropriate aromatic heterocyclic ring at the C-7 position of substituted camptothecin significantly enhances cytotoxicity. The compound **9c** (Figure 1) is thus modified in its structure 7-substituted 10,11-methylenedioxy-camptothecin via Suzuki reaction [24]. The new compound, with enhanced solubility, has been reported by us to have remarkable antitumor efficacy against drug-resistant small-cell lung cancer both in vivo and in vitro [24]. In another study, we discovered that **9c** exhibited more cytotoxic activity in the NSCLC cells, particularly in the A549 cells. Currently, there is a dearth of efficacious targeted drugs and immunotherapy agents for the disease modeled by this cell line.

Herein, we further probed into the potential inhibitory effects and underlying mechanisms of **9c** on the proliferation of NSCLC cells, mainly focusing on A549 cells, both in vitro and vivo. Our findings indicate that **9c** displays impressive antitumor effects in vitro and in vivo by destabilizing the NSA2-EGFR axis, thus blocking downstream signaling, while having no observable apparent toxicity in mice. More importantly, compound **9c** exhibits synergetic efficacy with AMG510, a well-known K-RAS^G12C^ inhibitor in the NCI-H358 xenograft mouse model.

## 2. Results

### 2.1. ***9c*** Inhibits the Proliferation of A549 and H1975 Cells by Arresting G2/M Phase

**Figure 2:**
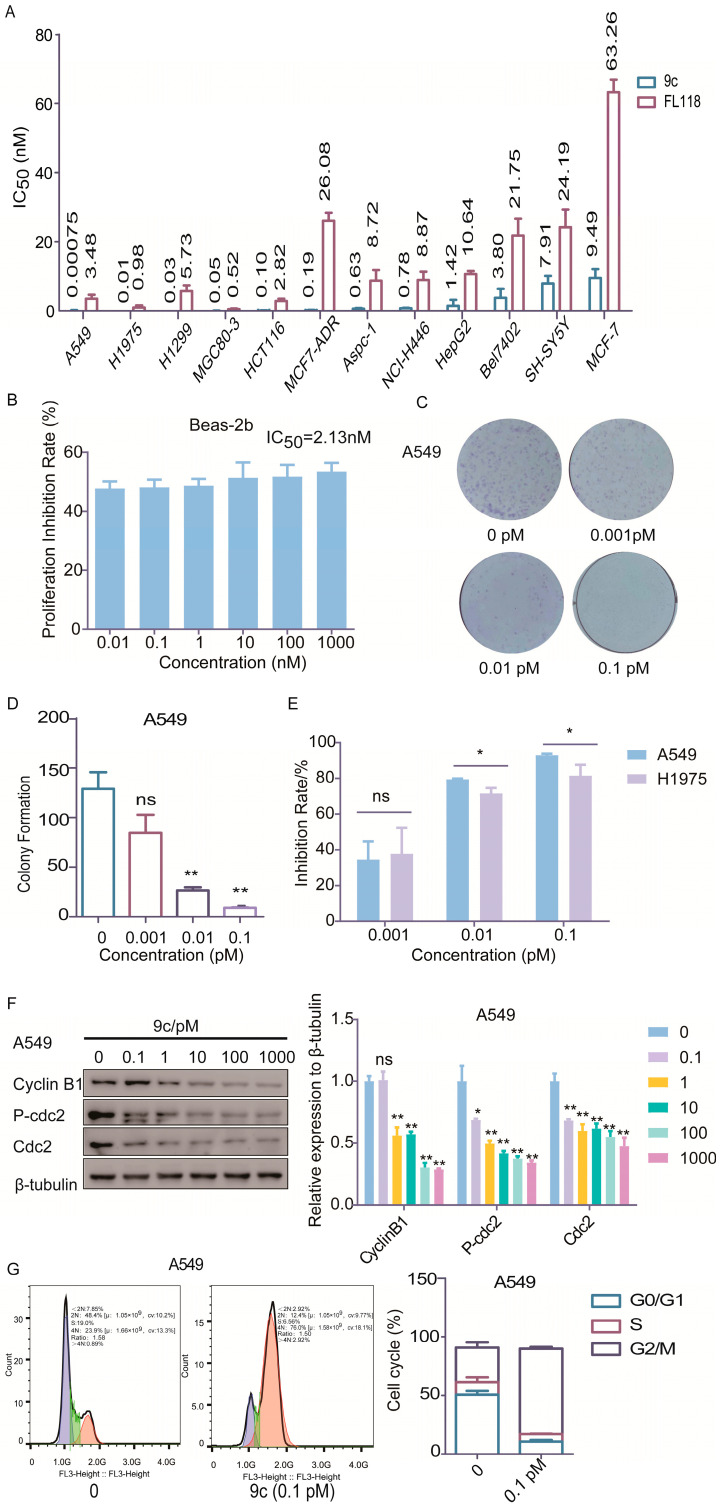
**9c** inhibits cell proliferation by blocking the cell cycle at the G2/M phase in A549 cells: (**A**) Various cancer cells are treated with indicated concentrations of **9c** for 72 h. Cell proliferation is determined using SRB and MTT assay. The data are presented as means ± SD from three independent experiments. (**B**) Proliferation inhibition of **9c** on Beas-2b cells. The cells are treated with **9c** for 72 h and detected by the CCK-8 method. (**C**) **9c** inhibits the colony formation. A549 cells are treated with (0.001–0.1 pM) **9c** for 14 d. One well plate scale = 35 mm. (**D**) The number of clones in plates is quantified. Data are shown as mean ± SD. ns, not statistically significant *p* > 0.05, * *p* < 0.05, ** *p* < 0.01. **9c** vs. control. (**E**) The inhibition rate of colonies. The inhibition rate of relative A549 vs. H1975. ns, not statistically significant *p* > 0.05, * *p* < 0.05, A549 vs. H1975. (**F**) Compound **9c** inhibits proteins related to G2/M phase cells. The cell cycle-related protein levels are assessed by Western blot analysis, with β-tubulin serving as a loading control. The densities of the protein band are quantified relative to β-tubulin (right panel). ns, not statistically significant *p* > 0.05, * *p* < 0.05, ** *p* < 0.01. **9c** vs. 0 pM group. (**G**) **9c** affects cell cycle distribution. The cell cycle distribution is performed on A549 cells after treatment with **9c** for 24 h. The DNA content is detected by flow cytometry.

The cytotoxic activities in vitro of compound **9c** against various human tumor cells were determined by SRB assay or MTT assay. As shown in Figure 2A, **9c** displayed quite varying inhibitory effects on different tumor cells following a 72 h treatment and had more significantly cytotoxic activity against NSCLC cells. A noteworthy aspect is that **9c** exhibited the most cytotoxicity against the A549 and H1975 cells with the IC50 = 0.75 pM and 10 pM, which was about 4640-fold or 98-fold more potent than **FL118**. Furthermore, the anti-tumor spectrum of **9c** is different from that of **FL118**, e.g., a certain type of cancer cell that is sensitive to **FL118** is not sensitive to **9c**. Notably, the cytotoxic effects of **9c** were significantly less pronounced on the normal cell line Beas-2b (the inhibition rate at 1000 nM was only 53.4%) than on cancer cells (Figure 2B).

The plate clone formation assay is an effective method for evaluating the growth ability of cancer cells in vitro [25]. Accordingly, **9c** significantly inhibited the colony-forming abilities of A549 and H1975 cells in a dose-dependent manner (Figure 2C,D and Figure S1A,B). Compared to the control group, the inhibition rates of A549 cells treated with **9c** (0.001–0.1 nM) for 14 days were 27.6%, 79.7%, and 93.0% in A549 cells, 37.8%, 71.7%, and 81.5% in H1975 cells, respectively, and the inhibitory potential on A549 cells was stronger than that observed on H1975 cells (Figure 2E). In order to explore whether the cell cycle arrest contributes to **9c**-induced proliferation inhibition, we further analyzed the cell cycle distribution upon **9c** treatment and found that **9c** was able to induce G2/M phase arrest in A549 cells. The proportion of G2/M phase cells in the control group was 23.9%, while it increased to 76.0% in the presence of 0.1 pM of **9c** (Figure 2G). Furthermore, the levels of Cyclin B1 and p-cdc2, essential for the control of the cell cycle at the G2/M (mitosis) transition, were repressed significantly in a dose-dependent manner (Figure 2F and Figure S1C). These results reveal that **9c** exerts a potent inhibitory effect on the proliferation of NSCLC cells by arresting the cell cycle at the G2/M phase.

### 2.2. ***9c*** Induces Cell Apoptosis of A549 Cells and H1975 Cells

**Figure 3:**
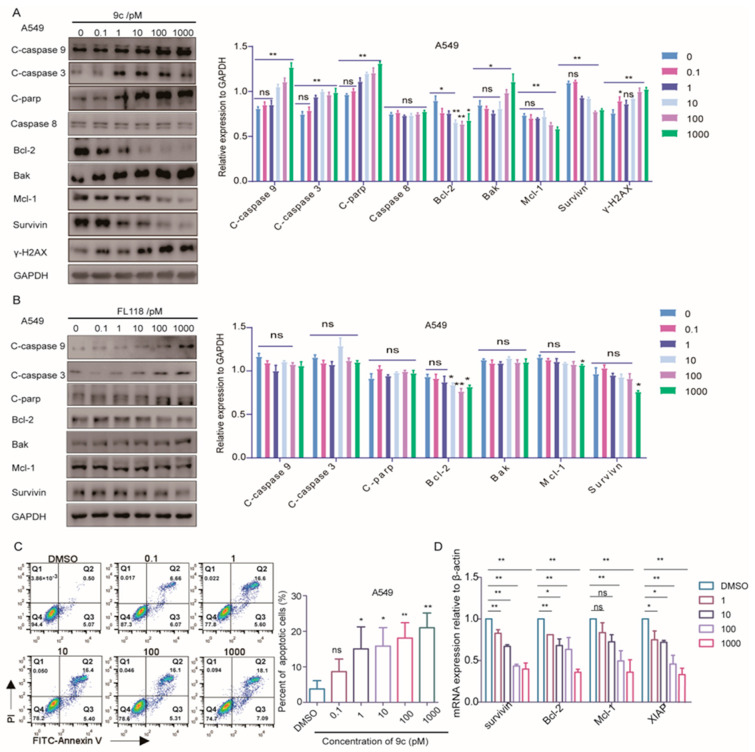
**9c** promotes the apoptosis of non-small cell lung cancer cell lines: (**A**,**B**) Western blot analysis of the effect of **9c** or FL118 on apoptosis-related proteins at 24 h. GAPDH is chosen as the internal control. The densities of the protein band are quantified relative to GAPDH (right panel). ns, not statistically significant *p* > 0.05, * *p* < 0.05, ** *p* < 0.01 vs. 0 pM group. (**C**) **9c** induces the apoptosis of cancer A549 cells. Apoptosis is analyzed by flow cytometry. A549 cells are treated with **9c** for 24 h, and apoptosis is analyzed by flow cytometry. Quantification of the Annexin-V positive cells (right panel). ns, not statistically significant *p* > 0.05, * *p* < 0.05, ** *p* < 0.01 vs. DMSO. (**D**) Upon exposure to **9c** (0–1000 pM) for 24 h, RT-qPCR is used to detect the mRNA levels of apoptotic molecules. ns, not statistically significant *p* > 0.05, * *p* < 0.05, ** *p* < 0.01 vs. DMSO.

Next, we determined whether G2/M phase arrest induced by **9c** resulted in cell apoptosis. Members of the caspase family of proteases play crucial roles in both the initiation and execution of apoptosis. Specifically, caspase-9 is typically the first caspase to be activated in response to apoptotic signals, while caspase-3 plays an essential role in apoptosis by cleaving PARP [26,27]. The ratio of Bax (an apoptosis promoter) to Bcl-2 (an apoptosis inhibitor), as a rheostat, was used to determine cell susceptibility to apoptosis [28,29]. As shown in Figure 3A and Figure S2A, the decrease in Bcl-2 expression was accompanied by the increase in Bak expression after **9c** treatment. We also confirmed that cleaved caspase-9, cleaved caspase-3, and cleaved-PARP were significantly upregulated after **9c** treatment in a dose-dependent manner. The levels of γ-H2AX, an critical marker of DNA double-strand breaks, was significantly elevated in a dose-dependent manner (Figure 3A), indicating that **9c** induced DNA double-strand breaks. Meanwhile, the levels of caspase-8, an essential component of the death signal receptor pathway of apoptosis [30], remained unchanged following exposure to **9c** treatment. Taken together, **9c** induced A549 cell and H1975 cell apoptosis involved in the mitochondrial pathway rather than the death receptor pathway. Furthermore, we conducted an apoptosis analysis using Annexin V-FITC/PI staining and found that **9c** facilitated a significant increase in the apoptosis rate of A549 cells. After a statistical analysis of the proportion of the apoptotic cells, it was observed that 0.1 pM, 1 pM, 10 pM, 100 pM, and 1000 pM **9c** caused 8.65 ± 2.89%, 15.03 ± 5.07%, 15.84 ± 4.21%, 18.08 ± 3.53% and 20.99 ± 3.39% apoptotic cells, respectively, compared with 3.75 ± 1.93% apoptotic cells in the control group (Figure 3C). Taken together, these data clearly indicate that **9c** suppresses the growth of A549 cells by G2/M phase arresting and then leads to cell apoptosis through the intrinsic mitochondrial pathway.

It was reported that **FL118** inhibits the transcription and expression of survivin, XIAP and Mcl-1 [13]; these protein play important roles in suppressing apoptosis. Then, we further examined whether **9c** has influences on the protein expression and mRNA transcription of these anti-apoptotic proteins (Figure 3D). Collectively, all these data illustrate that **9c** possesses the capacity to induce cell apoptosis by restraining the genetic transcription and expressions of multiple anti-apoptosis-related proteins, similar to **FL118**.

### 2.3. ***9c*** Inhibits Proliferation of A549 Cells Independent of Topo I Activitys

**Figure 4:**
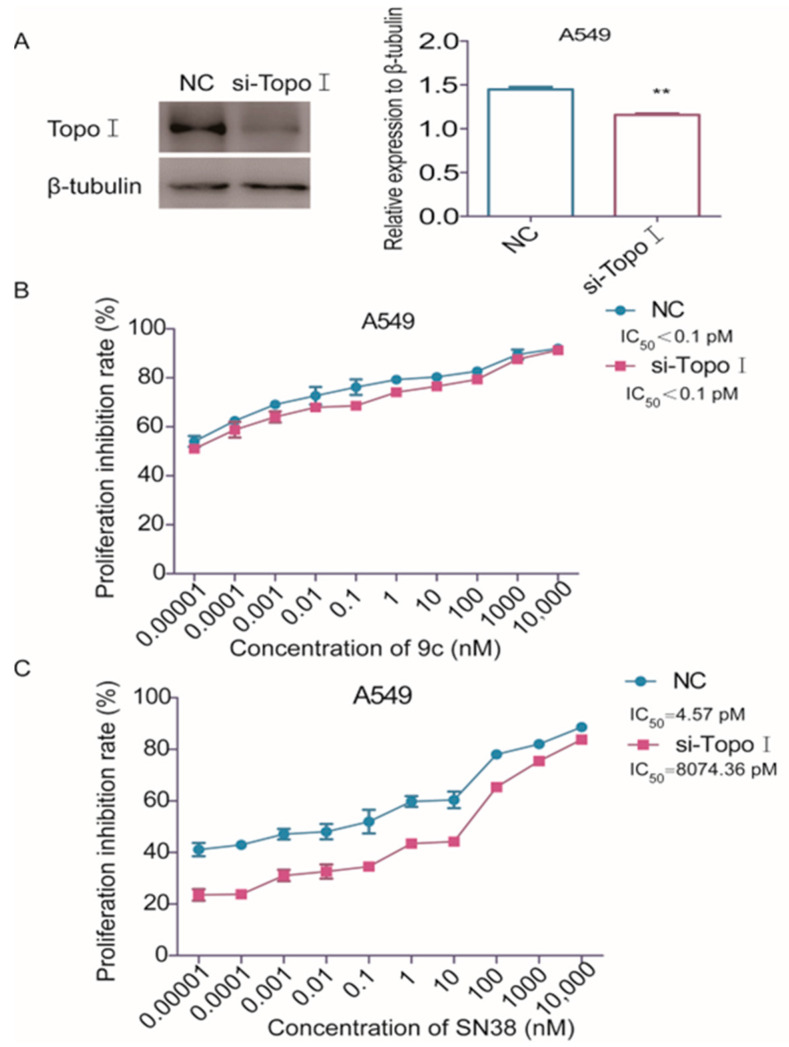
**9c** inhibits the proliferation of A549 cells independent of Topo I activity: (**A**) A549 cells are transfected with the Topo I targeting siRNA using Lipo3000 transfection reagents. After 48 h, the transfection efficiency is examined by Western blot. The protein level is assessed by Western blot analysis. The densities of the protein band are quantified relative to GAPDH (right panel) ** *p* < 0.01. si-Topo I vs. NC. (**B**,**C**) A549 cells with Topo I-knockdown are treated with indicated concentrations of **9c** or SN38 compounds for 72 h, and cell viability is determined by SRB assay.

DNA topoisomerase I (Topo I) had been firmly demonstrated to be the molecular target of the CPT class of compounds [31]. However, the inhibitory effect of **FL118** on topoisomerase I activity is not a primary determinant of its antitumor efficacy [13]. To ascertain whether **9c**, with a similar molecular structure to CPT, plays an antitumor role as an inhibitor of Topoisomerase I, we employed the small interference targeting deletion of Topo I (si-Topo I) in A549 cells and then detected the inhibitory activity of **9c** on the proliferation of A549 cells by SRB assay. The result showed that, after **9c** treatment for 72 h, the presence of siTopo I had no discernible impact on the IC_50_ value of A549 cells (Figure 4A,B). However, the metabolically active substance SN38 of irinotecan was revealed, a marked reduction in its ability to inhibit the proliferation of A549 cells after the deletion of Topo I, which is 1766-fold higher in the IC_50_ value than the negative control group (Figure 4C). Thus, our results suggest that compound **9c** exerts the suppression ability on the proliferation of A549 cells independent of Topo I activity.

### 2.4. Transcriptional and Proteomic Analysis of A549 Cells with or Without ***9c*** Treatment

**Figure 5:**
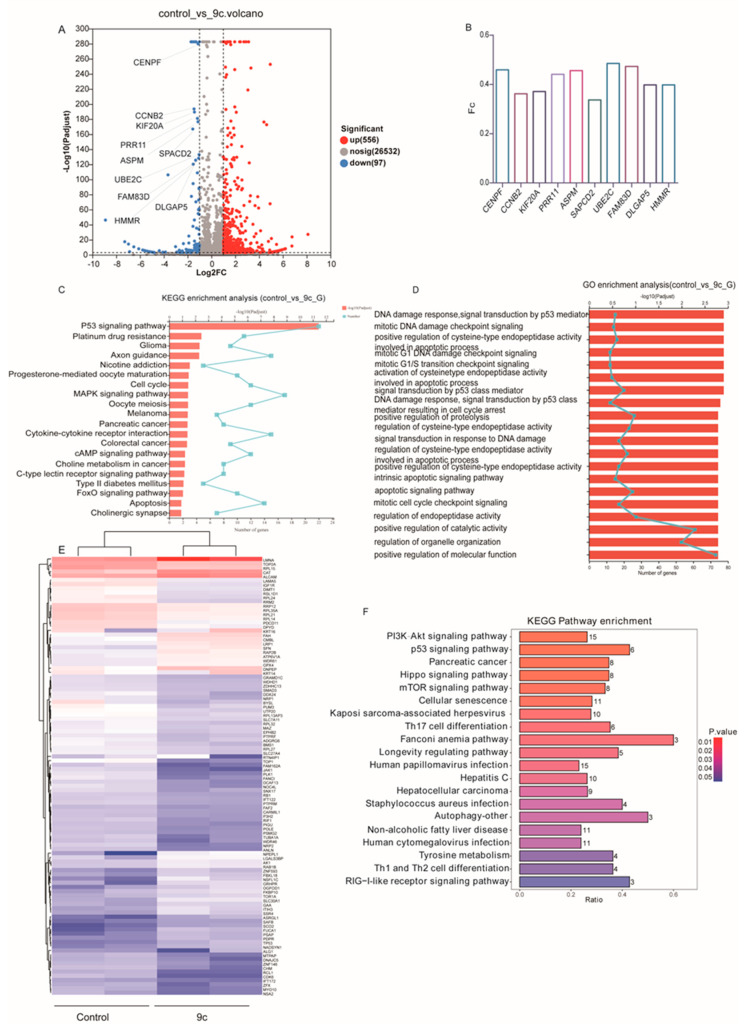
Association analysis of differential genes and differential proteins: (**A**) Volcano plot of differentially expressed genes between the **9c** group and the control group. (**B**) Top 10 downregulated genes in **9c** vs. control. (**C**,**D**) GO and KEGG enrichment analyses performed on the DEGs in A549 transcriptome. Column charts show the top 20 significantly enriched terms. (**E**) The cluster heat map in the A549 proteomics. The abscissa indicates the number of samples, whereas the ordinate indicates DEPs. The histogram in the upper right corner represents the color level; each rectangle corresponds to the expression value of a sample. (**F**) Column charts show the top 20 significantly enriched KEGG terms in the A549 proteomics.

To explore and investigate the mechanism by which **9c** elicits growth inhibition and cellular apoptosis, whole RNA sequencing was conducted to identify differentially DEGs by clustering the genes according to the similarity of gene expression profiles in **9c**-treated A549 cells compared with its counterpart. Our data revealed 653 differentially expressed genes shown as a volcano plot (Figure 5A). The top 10 genes with the most obvious down-regulation are shown in Figure 5B. These genes are related to the cell cycle and cell proliferation; for example, CCNB2 and CENPF modulates the G2/M phase and cell division [32,33], which is consistent with our results above, i.e., blocking the G2/M phase to inhibit cell proliferation by **9c**. Moreover, the DEGs were categorized based on cellular components (CCs), biological processes (BPs), and molecular functions (MFs). In the GO enrichment analysis, DEGs in **9c**-treated cells were mainly enriched in the DNA damage response signal transduction by the p53 mediator, mitotic DNA damage checkpoint signaling, and the positive regulation of cysteine-type endopeptidase activity and were involved in the apoptotic process (Figure 5D). The KEGG enrichment analysis revealed a strong association of the DEGs in the **9c** group with the regulation of the p53 signaling pathway, platinum drug resistance, and glioma and axon guidance (Figure 5C). Although transcription levels provide an overall indication of gene expression status, the post-translational regulation of RNA can impact the final abundance and activity of proteins to some extent. These effects may differ from those observed in a single transcription analysis. Therefore, we integrated a proteomic analysis to comprehensively elucidate the changes induced by **9c** in A549 cells. The expression of DEPs in **9c**-treated A549 cells was explored by clustering the proteins according to the similarity of proteins’ expression profiles of the samples. A heat map was generated to display the top 104 proteins with the smallest *p*-values, of which 44 proteins were up-regulated and 64 proteins were down-regulated in **9c**-treated A549 cells. Different protein expression levels were indicated by different colors. For instance, the colors from blue to white to red denote expression levels from low to high, where blue indicates lowly expressed proteins and red indicates highly expressed proteins (Figure 5E). The functions of these DEPs were explored through KEGG enrichment analysis. Results showed that the top five categories with the most significant enrichment in the **9c** group were largely associated with the PI3K-AKT signaling pathway, the p53 signaling pathway, pancreatic cancer, the Hippo signaling pathway, and the mTOR signaling pathway (Figure 5F). The aberrant activation of these signaling pathways is intricately associated with cell survival and proliferation, thereby contributing to the initiation and progression of cancer [31,32]. Correlation analysis between our transcriptome and proteome data yielded 22 overlapping genes, which were mainly enriched in the DNA damage, proliferation, and cell cycle in NSCLC cells (Table 1). Moreover, the p53 signaling pathway was identified through both data analyses. Collectively, these data support the view that **9c** exerts potent inhibitory effects on the proliferation, cell cycle, and other malignant processes of NSCLC by regulating the p53 signaling pathway and PI3K-AKT-mTOR signaling pathway.

### 2.5. ***9c*** Impairs the PI3K-AKT Pathway by Reducing the Stability of EGFRs

**Figure 6:**
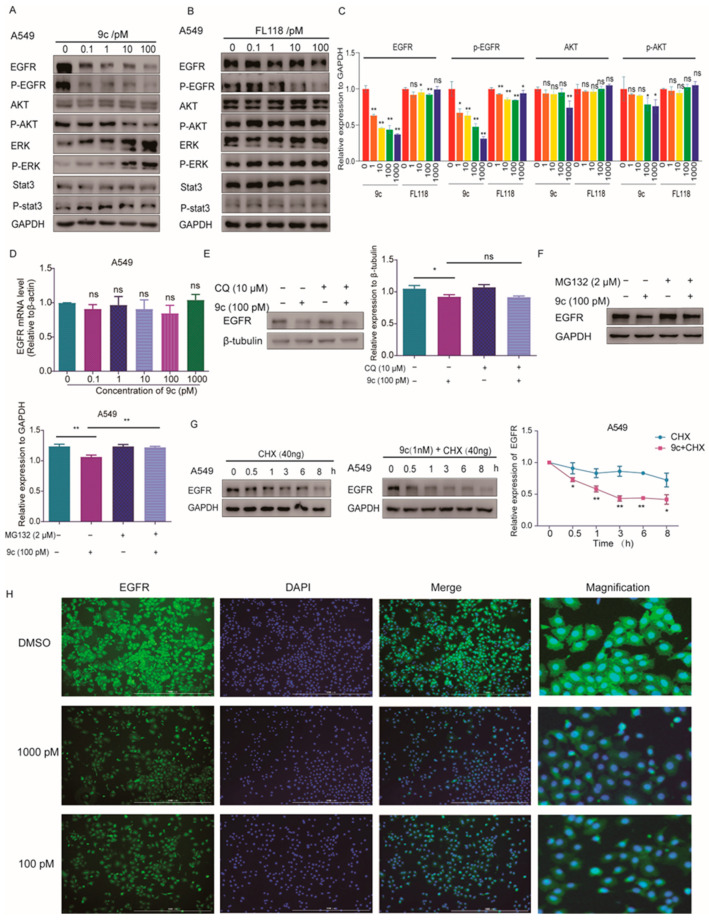
**9c** inhibits the EGFR-PI3K-AKT pathway: (**A**–**C**) The effects of **9c** on the expression levels of signaling molecules associated with proliferation. A549 cells are treated with **9c** (**A**) or FL118 (**B**) for 24 h. GAPDH is used as a loading control. (**C**) Protein band densities are quantified by normalizing to GAPDH. (**D**) A549 cells are treated with **9c** for 24 h. Then, the *EGFR* mRNA level is measured by quantitative-PCR. The quantitative PCR is run to quantify the mRNA level of the *EGFR*. (**E**,**F**) A549 cells are pretreated with proteasome inhibitor MG132 (2 μM) or lysosomal inhibitor Chloroquine (CQ, 10 μM) for 2h as indicated, and, then, 100 pM **9c** is added to cells for 15 h. The EGFR protein level is assessed by Western blot analysis, with β-tubulin or GAPDH serving as a loading control. The densities of the protein band are quantified relative to β-tubulin or GAPDH (right panel or lower panel). (**G**) A549 cells are treated with a 40ng /mL protein synthesis inhibitor cycloheximide (CHX) with or without 1 nM of **9c** for indicated lengths of time. The EGFR protein level was assessed by Western blot analysis. The densities of the protein band are quantified relative to GAPDH (right panel). (**H**) A549 cells are treated with **9c** for 24h. Cells are then stained anti-EGFR (labeling EGFR, green). Scale bar 1000 um. Data are presented as means ± SD. All experiments are performed in three replicates. ns, not statistically significant *p* > 0.05, * *p* < 0.05, ** *p* < 0.01 versus control.

Stat3, Akt and ERK1/2 are signaling molecules in the downstream three main signal pathways of the EGFR, which is abnormally activated in various cancer cells [34,35,36]. In proteomic analysis, the PI3K-AKT-mTOR pathway showed a notable alteration in the **9c** treatment group. Furthermore, we also discovered the reduced expression of the EGFR by 1.7-fold compared with the control group in the proteome data while not at the transcriptome level. We then examined the expressions and activations of these proteins as well as EGFRs by Western blot assay. As shown in Figure 6A and Figure S3A, after treatment with 0.1 pM, 1 pM, 10 pM and 100 pM of **9c** for 24 h, **9c** was confirmed to downregulate the protein level of EGFR in A549 and H1975 cells. The levels of stat3 and p-stat3 were not significantly changed, while the AKT and p-AKT were significantly decreased on A549 and H1975 cells in a concentration-dependent manner. On the contrary, p-ERK was markedly upregulated by **9c** (Figure 6A and Figure S3A). It is worth noting that the above alterations upon **9c** treatment were more pronounced than that of **FL118** (Figure 6C and Figure S3B). Together, these data indicate that **9c** exerts an anti-proliferative effect, potentially by impeding the EGFR-AKT pathway.

Subsequently, we sought to identify the specific mechanisms through which **9c** downregulated the EGFR. Firstly, we determined the impact of **9c** on the transcription of the EGFR to clarify the transcriptome data. It was revealed that there was no significant change with the mRNA level of the *EGFR* (Figure 6D), which is consistent with the transcription analysis. Therefore, it is considered that **9c** may have influenced the protein degradation in the cytoplasm. The principal pathways responsible for protein degradation in eukaryote cells encompass the ubiquitin-proteasome pathway and autophagy lysosome pathway [37]. Firstly, lysosome inhibitor chloroquine was used to detect whether EGFR protein was degraded by the lysosome pathway. The results showed that chloroquine treatment did not prevent the reduction in EGFR protein levels (Figure 6E). However, treatment with the protease inhibitor MG132 [38] significantly rescued the **9c**-induced downregulation of EGFR protein levels (Figure 6F). These results imply that **9c** probably causes the degradation of EGFR protein via the ubiquitin-proteasome pathway. Subsequently, we treated the cells with cycloheximide (CHX), an inhibitor of protein synthesis in eukaryotic cells, to investigate the stability of the EGFR in the presence of compound **9c**. As shown in Figure 6G compared with CHX treatment alone, the protein level of the EGFR decreased more expressively with the co-treatment of CHX and **9c**, indicating that **9c** shortens the lifetime of EGFR protein; that is, **9c** has the ability to destabilize EGFR protein. To ensure the degradation of the EGFR-affected membrane recruitment of the EGFR, an immunofluorescence assay was utilized and demonstrated that **9c** alleviated the abundance of EGFRs in the cell surface (Figure 6H). Overall, these results indicate that **9c** induces EGFR protein degradation and lessens its membrane content through the ubiquitin proteasome pathway.

### 2.6. Degradation of EGFR Upregulates p53 by ***9c*** Destabilizing NSA2

**Figure 7:**
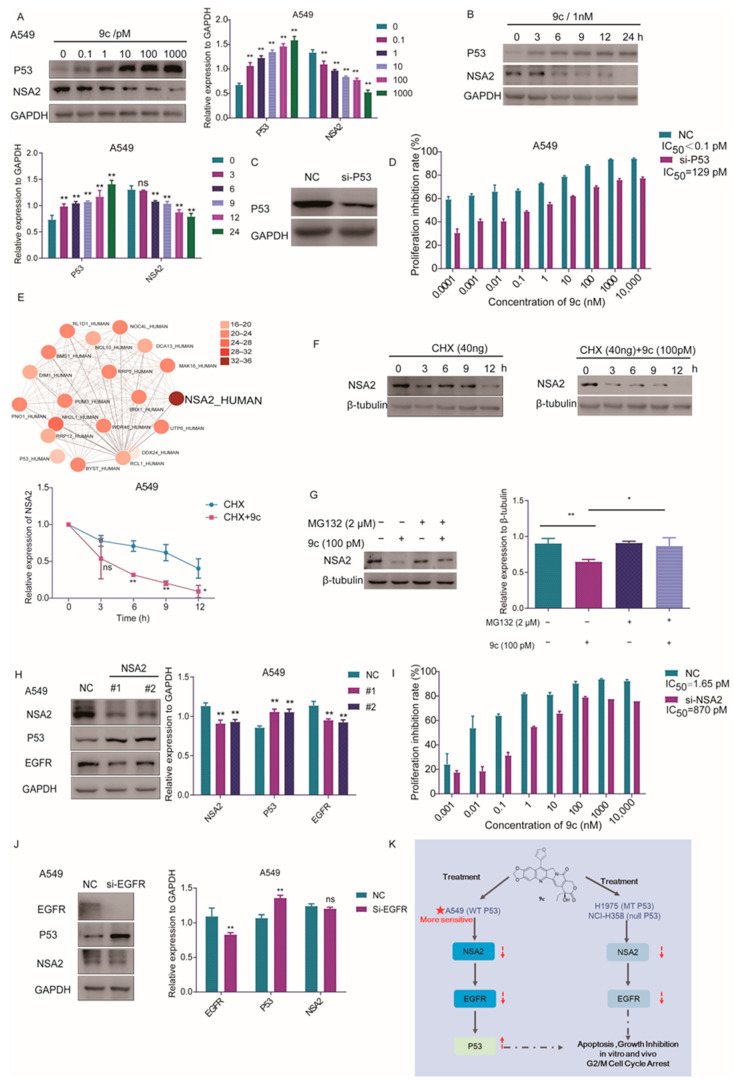
**9c** induced wild-type p53 expression by destabilizing NSA2-EGFR axis: (**A**,**B**) Effects of **9c** on the expressions of p53 and NSA2. A549 cells are treated with **9c** for indicated time. GAPDH is used as a loading control. (**C**) A549 cells are transfected with the p53 targeting siRNA using Lipo3000 transfection reagents. After 48 h, the transfection efficiency is examined by Western blot. (**D**) A549 cells with p53-knockdown are treated with indicated concentrations of **9c** compound for 72 h, and cell viability is determined by SRB assay. (**E**) The 20 proteins with the highest degree in the PPI relationship analysis of DEPs, and the color depth of the node represents the number of differentially expressed proteins that interacted with the protein. (**F**) A549 cells are treated with a 40ng/mL protein synthesis inhibitor cycloheximide (CHX) with or without 100 pM of **9c** for indicated lengths of time. NSA2 protein level is assessed by Western blot analysis, with β-tubulin serving as a loading control. The densities of the protein bands are quantified relative to β-tubulin (right panel). (**G**) A549 cells are pretreated with proteasome inhibitor MG132 (2 μM) for 2h as indicated, and then 100pM of **9c** was added to cells for 15 h. Cells are lysed, and NSA2 protein level are assessed by Western blot analysis, with β-tubulin serving as a loading control. The densities of the protein bands are quantified relative to β-tubulin (lower panel). (**H**) A549 cells are transfected with the NSA2 targeting siRNA using Lipo3000 transfection reagents. After 48 hours, protein levels are assessed by Western blot analysis, with GAPDH serving as a loading control. The densities of the protein bands are quantified relative to GAPDH (right panel). (**I**) A549 cells are transfected with the NSA2 targeting siRNA using Lipo3000 transfection reagents. After 48 h, the transfection efficiency is examined by Western blot. A549 cells with NSA2-knockdown are treated with indicated concentrations of **9c** for 72 h, and cell viability is determined by SRB assay. (**J**) A549 cells are transfected with the EGFR targeting siRNA using Lipo3000 transfection reagents. After 48 h, protein levels are assessed by Western blot analysis, with GAPDH serving as a loading control. The densities of the protein bands are quantified relative to GAPDH (right panel). (**K**) The schematic diagram of **9c** inhibiting the NSCLC progression. **9c** exerts a potential antitumor effect in non-small cell lung cancers in vitro and in vivo through the NSA2-EGFR signaling pathway. Data are presented as means ± SD. All experiments are performed in three replicates. ns, not statistically significant *p* > 0.05, * *p* < 0.05, ** *p* < 0.01 versus control.

The p53 signaling pathway was recognized significantly in both proteomic and genomic KEGG enrichment analysis. It has been reported that the knockout of EGFRs promotes the expression of wild-type p53 but has no significant effect on mutant p53 [39]. In the previous results, we demonstrated that **9c** induces the protein degradation of EGFRs, and, then, we determined the influence of **9c** on the protein expression level of p53. The result showed that wild-type p53 in A549 rather than mutant p53 in H1975 cells was elevated noticeably in a dose and time-dependent manner upon **9c** treatment (Figure 7A,B and Figure S4A). Naturally, in the p53-deficient cells, H358, no change was found in the p53 protein expression (Figure S4B). Furthermore, the cell proliferation ability was significantly alleviated in A549 cells with a knockdown of p53 upon **9c** treatment, and its IC_50_ value exhibited a 1290-fold increase compared to the control group (Figure 7C,D). These findings indicate that **9c** promotes p53 expression that is dependent on a decrease in EGFR protein levels, thereby enhancing the sensitivity of A549 to **9c** treatment. To elucidate how **9c** affects the EGFR-p53 signaling pathway, by mapping protein–protein interaction (PPI) networks of DEPs and their interactors, we identified a significant enrichment of NSA2 interactors (Figure 7E), suggesting that treatment with **9c** potentially hampers cell proliferation, probably by impacting NSA2. The overexpression of NSA2 markedly enhanced tumor cell proliferation, which may be attributable to the downregulation of p53 expression [40]. In the proteomic data, we observed that **9c** led to a significant decrease in the abundance of NSA2, which was confirmed by Western blot assay in A549 and H1975 cells in a dose-and time-dependent manner. In addition, the protein level of NSA2 was also found to be decreased in p53-deficient H358 cells at the same dose (Figure S4B). Because the NSA2 gene was not observed to be down-regulated in RNA sequencing analysis, we speculated that **9c** affected the stability of NSA2 protein. To demonstrate the hypothesis, A549 cells were studied in the presence of protein synthesis inhibitor CHX following treatment with **9c**. It was revealed that NSA2 was substantially destabilized by 55.2% as compared to CHX (Figure 7F). Moreover, the treatment with the protease inhibitor MG132 significantly rescued the **9c**-induced downregulation of NSA2 in protein levels (Figure 7G). These data suggest that **9c** abates the stability of NSA2 protein. Significantly, the knockdown of NSA2 decreased the EGFR protein level in p53 wild-type A549 cells, as well as in p53 mutant H1975 cells, but only increased p53 in A549 cells rather than in H1975 cells (Figure 7H and Figure S4C). Not only that, siNSA2 exhibited a more pronounced attenuation of the inhibitory effect of **9c** on cell proliferation in A549 cells compared to H1975 cells, with the decrease in IC50 value by 527.3-fold in A549 cells and 14.73-fold in H1975 cells (Figure 7I and Figure S4D). Moreover, we proved that siRNA targeting the EGFR enhanced the p53 expression in A549 cells and had no apparent impact on p53 mutant H1975 cells, which is in line with previous reports [39]. Meanwhile, no significant alteration in the NSA2 protein level was found in both A549 and H1975 cells (Figure 7J and Figure S4E). These results imply that **9c** exerts a proliferative inhibitory effect through the NSA2-EGFR signal pathway in p53 wild and p53 mutant cell lines. Collectively, all the findings indicate that **9c** induces the degradation of EGFRs by mitigating the stability of NSA2 and exhibits more potent cytotoxicity in the cancer cells with wild-type p53 by upregulating p53 due to the degradation of EGFRs. Together, these data suggest that **9c** exerts its anti-tumor effects mainly through inhibition of the NSA2-EGFR signaling pathway (Figure 7K).

### 2.7. ***9c*** Suppresses the Growth of A549 Xenograft In Vivo

**Figure 8:**
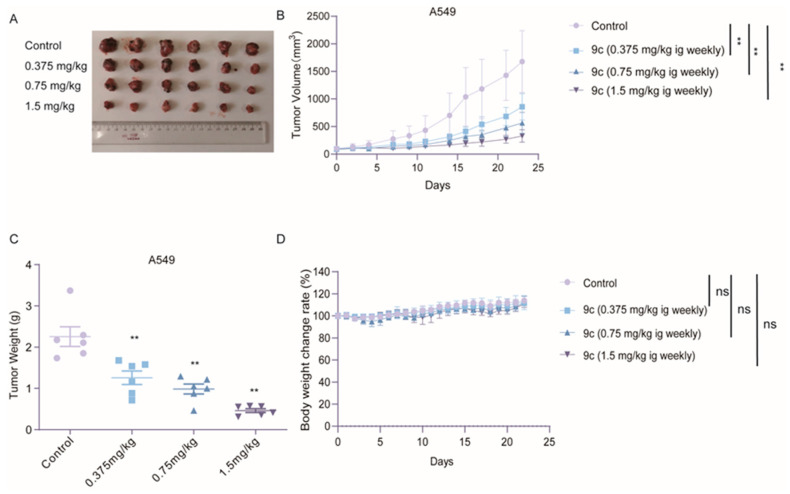
**9c** inhibits A549 xenograft growth in vivo: (**A**–**D**) A549 cells are inoculated into the right flank of Balb/c-nude mice. Once the tumor nodules reach a volume of 100 mm³, the animals are randomly allocated to four groups and, respectively, intragastrically injected with the control (*n* = 6), **9c**, 0.375 mg/kg (*n* = 6), 0.75 mg/kg (*n* = 6), and 1.5 mg/kg (*n* = 6). After 23 days of treatment, mice are sacrificed and tumors are harvested. The tumor image is photographed (**A**,**B**). The tumor volume is measured every 2 days. (**C**) Tumors are weighed, and the mice body weights (**D**) are measured every day. Data are presented as mean ± S.D. (*n* = 6); ns, not statistically significant, *p* > 0.05; ** *p* < 0.01, compared with the control group.

To observe that the efficacy of in vitro can be validated within the physiological context, a study was conducted utilizing the A549 xenograft model. **9c** was given at various doses by intragastric administration once per week upon reaching a tumor volume of 100 mm^3^. The results are illustrated in Figure 8. The tumor volume exhibited a significant dose-dependent reduction in the **9c** treatment group (Figure 8B). At the end of the experiment, tumor tissue is dissected and photographed (Figure 8A). The weights of both the tumor tissues and mice were measured. **9c** significantly suppressed tumor growth, with tumor growth inhibitory rates of 44.22% (0.375 mg/kg), 56.33% (0.75 mg/kg), and 79.57% (1.5 mg/kg) in a dose-dependent manner, respectively (Figure 8C). Fortunately, the nude mice bodies weighed at the indicated dose of the **9c** group were not obviously affected compared with control group (Figure 8D). These findings indicate that **9c** has the capacity to repress non-small cell lung cancer growth in vivo with weak toxicity.

### 2.8. ***9c*** in Combination with AMG510 Exerts Synergistic Antitumor Effects

**Figure 9:**
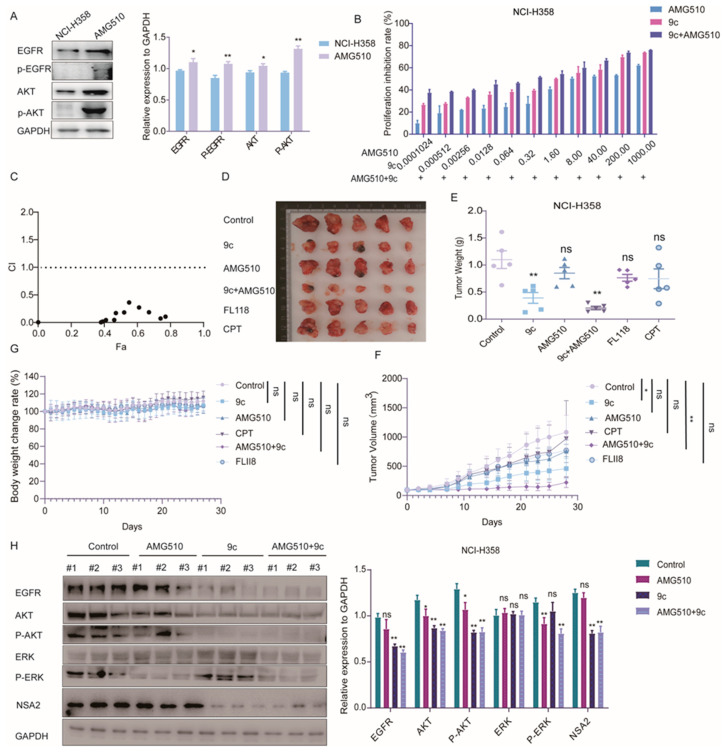
**9c** in combination with AMG510 exerts antitumor effects in vitro and vivo: (**A**) Western blot detected the differences in the EGFR and AKT between H358 parental and NCI-H358 AMG510 resistant strains. GAPDH was used as a loading control. Protein band densities are quantified by normalizing to GAPDH (right panel). (**B**) NCI-H358 cells are treated with indicated concentrations of **9c** or AMG510 for 72 h. Cell proliferation is determined using SRB assay. The data are presented as means ± SD from three independent experiments. (**C**) The representative combination indices (CIs) of **9c** (T) in combination with AMG510: CI < 0.9 signifies synergy, CI ranging from 0.9 to 1.1 indicates additivity a, and CI > 1.1 suggests antagonism. (**D**–**G**) NCI-H358 cells are injected into the right flank of Balb/c-nude mice. When the volumes of tumor nodules reached 100 mm^3^, the mice are randomly assigned to indicated groups and, respectively, intragastrically (ig) or intraperitoneally (ip) injected with the control (*n* = 5), 0.75 mg/kg of CPT (*n* = 5), 0.75 mg/kg of **9c** (*n* = 5), 0.75 mg/kg of FL118, 3 mg/kg of AMG510, and the combination (**9c** + AMG510, *n* = 5). After 28 days of treatment, mice are sacrificed and tumors are harvested. (**D,F**)The tumor image is photographed, (**D**) and the tumor volumes (**F**) are measured every 2 days. (**E**) Tumors are weighed, and the mice body weight (**G**) are measured every day. (**H**) Effect of **9c** on the expressions of proteins in tumors. Tumors are excised and lysed. Protein levels are assessed by Western blot analysis, with GAPDH serving as a loading control. The densities of the protein bands are quantified relative to GAPDH (right panel). Data are presented as mean ± s.d. (*n* = 5); ns, not statistically significant *p* > 0.05; * *p* < 0.05; ** *p* < 0.01; compared with the control group.

The combination of conventional chemotherapy drugs and novel drugs is often considered to increase the benefit to patients. AMG510 is an orally active, first-in-class, small molecule inhibitor targeting against K-RAS^G12C^, which inhibits the augmented activation of the RAS-ERK1/2 signaling pathway resulting from mutants of RAS. However, AMG510 leads to the activation of EGFR and PI3K-AKT pathways at advanced stages during treatment, thereby attenuating the efficacy and ultimately resulting in resistance [41]. As mentioned above, **9c** facilitates the degradation of the EGFR and blocks the PI3K-AKT signaling pathway. Therefore, the combination treatment **9c** with AMG510 may reinforce the antitumor efficacy of AMG510. Firstly, we examined the inhibitory proliferation ability of the co-administration of them in the H358 cell with the K-RAS^G12C^ mutant, we found that the cells up-regulated the expressions of the EGFR, p-EGFR, AKT and p-AKT after AMG510 treatment (Figure 9A), and we discovered that the combination had a synergistic effect on the cell growth of NCI-H358 cells, with the combination indices (CIs) less than 0.9 (Figure 9B,C). Subsequently, the in vivo effects of AMG510 and **9c** combination therapy against NCI-H358 xenografts were assessed. Nude mice were inoculated with NCI-H358 cells and then received individual or combined drug treatments via intraperitoneal injection when the tumor volume reached 100 mm^3^. It was shown that neither AMG510 nor **FL118** alone displayed significant efficacy against the tumor at the indicated dosage. In addition, CPT alone had no significant effectiveness on tumor progress at a dose of 0.75 mg/kg similar to **9c**. Meanwhile, **9c** was unveiled to repress the tumor growth with the inhibitory rate at 64.38% Furthermore, as anticipated, the pilot combination therapy (AMG510+**9c**) resulted in dramatically reduced tumor growths, with tumor growth inhibition rates up to 81.38% compared with control mice, and exhibited superiority over AMG510 monotherapy (Figure 9D–F). With these doses, all the mice in the treatment groups survived during the experiments. The body weight of mice treated with the combination of AMG510 and **9c** was not reduced during 4 weeks after the treatment, indicating the negligible toxicity in the combination therapy (Figure 9G). At the end of the experiment, tumors were isolated, and signaling proteins were detected by Western blot. **9c** had the capability to decrease the protein levels of the EGFR, p-AKT, P-ERK and NSA2 in tumor tissue from NCI-H358 xenografts (Figure 9H). Altogether, the combination therapy with AMG510 and **9c** had superior anti-tumor benefits in K-RAS ^G12C^ mutant NSCLC.

## 3. Discussion

In the presence of this paper, we provide evidence to demonstrate that **9c**, a novel camptothecin derivatives, exhibits impressively potential in combating the growth of NSCLC without known specific molecular targets in vitro and in vivo with efficacy superior to **FL118**, a well-known camptothecin analog. These above effects may be achieved by destabilizing the NSA2-EGFR axis by **9c**.

Our data showed that **9c** suppressed the proliferation of A549 and H1975 cells by arresting the cell cycle at the G2/M phase, thereby resulting in cell apoptosis. Furthermore, **9c** induced apoptosis involved in the mitochondrial pathway by regulating transcriptions and expressions of multiple anti-apoptosis-related proteins, such as Mcl-1, Bcl-2, XIAP, and survivin, with a similar effect as **FL118**. Additionally, comparable to **FL118**, **9c** was also observed to repress the proliferation of A549 cells independent of Topo I activity. Meanwhile, the anti-tumor spectrum of **9c** was by no means different from that of **FL118**. CPT and its analogs target topoisomerase I (Topo I) as their therapeutic mechanism. In contrast, the sensitivity of human cancer cells or tumors to **FL118** is independent of Topo I expression, similar to the behavior observed with **9c**. Notably, **FL118** inhibits cancer cell growth at concentrations ranging from high pM to nM, whereas its inhibition of Topo I activity occurs at micromolar μM levels [13]. The findings suggest that the **9c** may act on alternative molecules in cancer cells.

Over the past years, intensive medicinal chemistry efforts have generated numerous CPT derivatives, and the variety of biological activities and medicinal applications exhibited by CPT analogs are impressive. It has already been noted that CPT may affect cellular function at loci other than Topo I, for example, functions as the inhibitor of acetylcholinesterase activity and HIF-1 [42]. **FL118** has been reported to strongly bind to and degrade the DDX5 oncoprotein via the proteasome degradation pathway without decreasing DDX5 mRNA, thereby controlling the expression of multiple oncogenic proteins, including survivin, Mcl-1, XIAP, Ciap [43]. Additionally, **FL118** was also suggested to inhibit p53 ubiquitination by blocking Mdm2–MdmX E3 complex formation via the targeting degradation of MdmX. Herein, we clarified that **9c** induced the degradation of EGFRs by mitigating the stability of NSA2. All together, we speculate that **FL118** and its derivatives can actually act as a ’molecular glue degrader’ to directly glue some protein and ubiquitination regulators together to degrade these protein, and which protein will be glued is highly dependent on the derivative primary structure and steric configuration although sharing the same core structure as them.

The frequent overexpression and/or mutation of EGFRs in lung cancer play an active role in facilitating cell proliferation [44,45], and EGFR-AKT, EGFR-STAT and EGFR-ERK signaling pathways play crucial roles in regulating various cellular processes essential for growth and survival. The abnormalities of these pathways have been extensively documented in numerous human tumors, and inhibitors against these pathways promote cell apoptosis [46,47]. We found that **9c** distinctly decreased the protein levels of EGFRs through the ubiquitin-proteasome pathway thorough destabilizing NSA2 and subsequently inhibiting the down-streaming signaling pathway of EGFR-AKT. The advantage of EGFR degradation reduces the possibility of its secondary mutation. Surprisingly, compound **9c** heightened the activation of ERK, probably due to the activation of alternative pathways in tumor cells resulting from the potent inhibition of EGFRs, as it has reported that the EGFR inhibitor causes the activation of ERK signaling mediated by SRC bypass [48]. The above characteristics of **9c** prompted us to evaluate antitumor effects of the combination of **9c** and AMG510, and the desired synergetic outcome has been attained. It has been reported that the upstream activation of several RTKs interferes with the KRAS^G12C^ blockade, and EGFR signaling appears to be a predominant mechanism underlying colorectal cancer resistance to AMG510 [49]. In our study, we found that the activation of the EGFR-AKT pathway occurred in the early treatment of AMG510. Therefore, **9c** in combination with AMG510 probably impedes the resistance occurrence upon AMG510 therapy. Our findings present a significant application of **9c** in the context of clinical combination therapy with a KRAS inhibitor in the future.

The tumor suppressor protein p53 serves as a stress-responsive transcription factor and a key determinant of cancer therapy responses [50,51]. p53 is activated in response to chemotherapy, radiation, and other DNA-damaging stresses. The activation of p53 leads to the repression of CDKs and cyclin B, which are essential for mitotic entry and contribute to G2/M phase arrest [52,53]. In addition, p53 binds physically to anti-apoptotic proteins (Bcl-2, Bcl-xL and Mcl-1) and inhibits the transcriptions and expressions of them, thereby indirectly inducing apoptosis [54]. The present results of **9c** are in line with these reports. P53 is a wild-type in  50% of non-small cell lung cancers (NSCLCs) [51]. It has reported that **FL118** exhibits cytotoxic effects in both a p53-independent and p53-dependent manner. Consistently, **9c** demonstrated potent cytotoxicity against both wild-type and mutant p53 cancer cells. p53 wild-type status has been associated with improved therapy responses and better outcomes in NSCLCs in several studies [55,56,57]. Not surprisingly, **9c** exhibits more potent cytotoxicity in the cancer cells with wild-type p53.

NSA2 is highly expressed in a variety of tumor cells, such as A549 and HepG2 cells [58]. However, the role of NSA2 in cancer development remains obscure. In this present study, **9c** can be regarded as a molecular probe, and we disclose for the first time that NSA2 functions as a stabilizer of EGFR protein, in turn recruiting more EGFRs onto the cell membrane to enhance the PI3K-AKT proliferative signaling pathway along with the down-regulation of p53 in the wild-p53 cancer cells, which provides the possible mechanism of NSA2 in the cancer progress. Further research is required to demonstrate how **9c** promotes NSA2 degradation, and the mechanism by which NSA2 induces the degradation of EGFRs remains to be elucidated.

## 4. Materials and Methods

### 4.1. Cell Culture

Human cell line MGC80-3, HCT116, ASPC-1, HepG2, SH-SY5Y, A549, H1299, Beas-2b, Bel7402, H1975, NCI-H358 and NCI-H446 were provided by the Institute of Biochemistry and Cell Biology, the Chinese Academy of Sciences (Shanghai, China). MCF-7, (Adriamycin-resistant) MCF-7/ADR, NCI-H446, MGC80-3, H1975, Beas-2b, NCI-H358, Bel7402, and ASPC-1 were cultured in 1640 medium (Gibco, Grand Island, NY, USA), which was supplemented with 10% fetal bovine serum, an additional 25 mg/L of glucose, and 1 mM of sodium pyruvate (Solarbio, Beijing, China). HCT116 and SH-SY5Y were cultured in Dulbecco’s Modified Eagle Medium supplemented with 10% fetal bovine serum. HepG2 was maintained in Minimum Essential Medium supplemented with 10% fetal bovine serum. A549 cells were cultured in F-12K medium supplemented with 10% fetal bovine serum. All cells were grown to confluence at 37 °C in a humidified atmosphere with 5% CO_2_.

### 4.2. Cell Transfection

si-Topo I, si-TP53, si-EGFR, si-NSA2 and the scrambled negative control were purchased from GenePharma (Shanghai, China). Cells were seeded in 6-well plates, incubated overnight, and then transiently transfected with siRNA and the negative control using Lipofectamine 3000 (Invitrogen, Inc., Waltham, MA, USA) according to the manufacturer’s instructions. Si-Topo I targets human Topo I at 5′-GAAAGGAAAUGACUAAUGAUU-3′, si-P53 at 5′-GACUCCAGUGGUAAUCUACTT-3′. si-EGFR at 5′-GAGGCAAAGUGCCUAUCAATT-3′.si-NSA2 #1 GAGCUAAAGUACUUUCCAATT, #2 GGGUGUUAUUACCAAAGGUTT. As a control, we used the negative-control siRNA from GenePharma (GenePharma Co., Ltd., Shanghai, China)

### 4.3. Extraction of Total RNA and RT-qPCR

Total RNA was extracted with the Trizol reagent (Invitrogen, Waltham, MA, USA). cDNA was synthesized with M-MLV reverse transcriptase (Promega, Fitchburg, WI, USA) and quantified by real-time qPCR using a Biosystems StepOne™ Real-Time PCR system and Fast SYBR Green Master Mix (Applied Biosystems, Foster, CA, USA), with β-actin as internal controls. PCR primers were designed with the NCBI online software Primer-BLAST (https://www.ncbi.nlm.nih.gov/tools/primer-blast/index.cgi?LINK_LOC=BlastHome, accessed on 16 May 2023) and synthesized by Tsingke (Tsingke Biotechnology Co., Ltd., Beijing, China). The PCR conditions were as follows: 94 °C for 2 min, followed by 30 cycles of 94 °C for 30 s, 60 °C for 30 s and 72 °C for 1 min, and 72 °C for 10 min. The primer sequences were as follows: β-actin, forward 5′ CGAGATCCCTCCAAAATCAA 3′; and reverse, 5′ TTCACACCCATGACGAACAT 3′, survivin, forward 5′ TGGCGTA AGATGATGGA 3′; and reverse, 5′ TAGGGACGACGATGAAA 3′, XIAP, forward 5′ AACACGTACTTGTGCG 3′; and reverse, 5′ ACTTTGATCTGGCTCA 3′, Mcl-1, forward 5′ AAAGCCTGTCTGCCAAAT 3′; and reverse, 5′ TATAAACCCACCACT CCC 3′, Bcl-2, forward 5′ GCCTTCTTTGAGTTCG 3′; and reverse, 5′ CAGCCTC CGTTATCC 3′, EGFR, forward 5′-TCTCAGCAACATGTCGATGG-3′ reverse, 5′-TCGCACTTCTTACACTTGCC-3′.

### 4.4. Western Blot

Cells were lysed in cell lysis buffer for Western blot (Beyotime Biotechnology, Shanghai, China) supplemented with a protease inhibitor PMSF at 4 °C for 30 min. Protein concentrations of the lysates were measured using the BCA reagent (Beyotime Biotechnology). Equal amounts of protein were run on 10% SDS-PAGE gels, then transferred to nitrocellulose membranes (Pall), and probed with the indicated primary antibodies. Anti-rabbit and anti-mouse (1:5000) HRP-conjugated secondary antibodies were used. Blots were detected by chemiluminescence with the enhanced chemiluminescence detection reagents (PIERCE, Rockford, IL, USA). The primary antibodies included GAPDH (Abclonal, Wuhan, China, AC002), β-tubulin (Abclonal, China, AC021), Cyclin B1 (12231), cdc2 (77055), p-cdc2 (2543), Cleaved Caspase-3 (9664), Cleaved Caspase-9 (9505), Cleaved PARP (5625), Bak (12105), Bcl-2 (4223), Mcl-1 (5453), survivin (2808), γ-H2AX (2577), stat3 (12640), p-stat3 (9145), erk1/2 (4695), p-erk1/2 (9101), AKT (4691), and p-AKT (4060), EGFR (4267), p-EGFR (3777) (Cell Signaling Technology, Boston, MA, USA), NSA2 (ER61622) and p53 (ET1601-13) (HUABIO, Hangzhou, China). CPT and AMG510 were purchased from MedChemExpress (Hoboken, NJ, USA).

### 4.5. Cell Proliferation Assay

SRB assays, an MTT assay, and a CCK-8 assay were used to measure the inhibition of cancer cell proliferation. For the SRB assay, adherent cells were seeded in 96-well plates (5000 cells/well) and treated with various concentrations of the indicated samples. After 72 h, the cells are immobilized with TCA and tinted with an SRB stain. The SRB stain that bound to cells was dissolved and quantitatively analyzed at a wavelength of 515 nm. For the MTT assay, MTT was added and incubated at 37 °C for another 4 h. The formazan product was dissolved and quantitated by spectrophotometry at a wavelength of 570 nm. The cytotoxicity of compounds was expressed as IC50. For the CCK-8 assay, CCK-8 was added and incubated at 37 °C for another 1 h, and the CCK-8 was quantitatively analyzed at a wavelength of 450 nm. All experiments were repeated at least three times.

### 4.6. Colony Formation Assay

A549 and H1975 cells were plated into 6-well plates at a density of 500 cells per well, treated with various concentrations of **9c** for 2 weeks. Then, cells were fixed in methanol at room temperature for 3 min and immersed in Giemsa for 15 min. Finally, the colonies were counted and photographed.

### 4.7. Flow Cytometry for Cell Apoptosis

The apoptosis was performed using the Annexin V-FITC/PI apoptosis detection kit (Absin Bioscience Inc., Shanghai, China) according to the manufacturer’s instructions. In brief, 1 × 10^6^ cells were incubated with various concentrations of **9c** for 24 h, then harvested by centrifugation, and washed with PBS twice. Cells were resuspended in a 100 μL binding buffer containing Annexin V-FITC (5 μL) and PI (5 μL) followed by incubation for 10 min at room temperature in the dark. Before flow cytometric analysis, 400 μL of binding buffer was added. After filtration, cells were analyzed by flow cytometry (MFLO XDP; Beckman Coulter, Brea, CA, USA).

### 4.8. Immunofluorescence Assay

A549 cells were permeabilized with 0.1% Triton X-100 in PBS and blocked with 1% bovine serum albumin followed by incubation with the indicated antibodies overnight. Cells were then stained with secondary antibodies. 4,6-Diamidino-2-phenylindole was used to visualize the nuclei.

### 4.9. RNA-Seq Analysis

Total RNA was extracted from the tissue using the TRIzol^®^ Reagent according to the Manufacturer instructions. Then, RNA quality was determined by a 5300 Bioanalyzer (Agilent, Santa Clara, CA, USA) and quantified using the ND-2000 (NanoDrop Technologies, Wilmington, DE, USA). RNA purification, reverse transcription, library construction, and sequencing were performed at Shanghai Majorbio Bio-pharm Biotechnology Co., Ltd. (Shanghai, China) according to the manufacturer’s instructions (Illumina, San Diego, CA, USA). The XX RNA-seq transcriptome library was prepared following Illumina^®^ Stranded mRNA Prep, Ligation from Illumina (San Diego, CA, USA), using 1 μg of total RNA.

### 4.10. Proteomic Analysis

In total, 300 μL of 8M urea was added to the sample, and the protease inhibitor was added at 10% of the lysate. After centrifuging at 14,100× *g* for 20 min, the supernatant was collected. The protein concentration was determined using the Bradford method, and rest was frozen to −80 °C. The proteins were identified by the LC–MS/MS approach (Qinglian Biotech Co., Ltd., Beijing, China). All RAW files were analyzed using the Proteome Discoverer suite (version 2.4, Thermo Fisher Scientific, Waltham, MA, USA).

### 4.11. Cell Cycle Analysis

A549 cells were incubated with various concentrations of **9c** for 24 h. After that, the cells were collected and washed in PBS and fixed in ice-cold 70% (*v*/*v*) ethanol overnight at −20 °C. Cells were resuspended in PBS and stained with a mixture containing RNase (10 μg/mL) and PI (50 μg/mL) for 20 min in the dark. After filtration, cells were analyzed by flow cytometry (MFLO XDP; Beckman Coulter, Pasadena, CA, USA).

### 4.12. In Vivo Study

Male BALB/c-nu mice aged 4–6 weeks were purchased from Charles River. The mice were housed in a pathogen-free animal facility and randomly assigned to the control or experimental group. For each cell line, 8 × 10^6^ A549 or NCI-H358 cells were resuspended in 200 μL of PBS and then subcutaneously injected into the BALB/c-nu mice. Tumor formation was monitored every three or four days by measuring the largest and smallest diameter of the formed tumors. The tumor volumes of these mice were checked to assess tumor growth. When the tumors reached about 100 mm^3^, the tumor-bearing mice were randomly allocated to various experimental groups and treated by intragastric administration with different drugs. When the tumor volume reached about 1500 mm^3^, tumors were excised, weighed, ground, and disrupted on ice for 30 min in the loading buffer, and, then, they were boiled for 15 min. Protein levels were analyzed by Western blot. Animal care practices and all experiments were reviewed and approved by the Committee on the Ethics of Animal Experiments of Ocean University of China.

### 4.13. Statistical Analysis

All statistical analyses were performed using SPSS 17.0 (SPSS, Chicago, IL, USA) with one-way ANOVA. All data are presented as the mean ± S.E.M. A *p* value less than 0.05 is indicated with *, and a value less than 0.01 is indicated with **.

## 5. Conclusions

In summary, **9c** exerts a potential antitumor effect in non-small cell lung cancers in vitro and in vivo through the NSA2-EGFR signaling pathway, which is more effective in p53-wild-type NSCLCs. Moreover, **9c** has the capability to significantly improve the antitumor effect of AMG510. Our finding indicates that **9c** is a promising new CPT derivative worthy of studying in the future.

## Figures and Tables

**Table 1:** Representative genes differentially expressed in the A549 and **9c**-treated A549 at the mRNA and protein levels.

mRNA Information	Protein Information	mRNA Sig *p*-Value	Protein Sig *p*-Value	Regulation Type (mRNA/Protein)
ZNF385A	Q96PM9	yes	yes	Up/Down
TP53I3	Q53FA7	yes	No	Up/Up
TM7SF2	O76062	yes	No	Up/Up
TAP1	Q03518	yes	yes	Up/Up
SFN	P31947	yes	yes	Up/Up
RRM2B	Q7LG56	yes	No	Up/Down
PTGS2	P35354	yes	No	Up/Down
PLK1	P53350	yes	yes	Down/Down
KPNA2	P52292	yes	No	Down/Down
HSPA4L	O95757	yes	No	Up/Up
HRNR	Q86YZ3	yes	yes	Up/Up
H2AC20	Q16777	yes	yes	Down/Down
FBXO22	Q8NEZ5	yes	yes	Up/Up
EML2	O95834	yes	No	Up/Up
DPYSL4	O14531	yes	yes	Up/Up
DDB2	Q92466	yes	No	Up/Up
CYFIP2	Q96F07	yes	yes	Up/Up
CMBL	Q96DG6	yes	No	Up/Up
BOLA2	Q9H3K6	yes	No	Down/Up
AS3MT	Q9HBK9	yes	No	Up/No
ARL6IP1	Q15041	yes	yes	Down/Up
APOBEC3C	Q9NRW3	yes	up	Up/Up

## Data Availability

Data are contained within this article or the Supplementary Materials.

## Supplementary Materials

The following supporting information can be downloaded at https://www.mdpi.com/article/10.3390/ijms26051987/s1.

## Abbreviations

The following abbreviations are used in this manuscript:

MCL-1	Myeloid cell leukemia 1
Bcl-2	B-cell lymphoma-2
XIAP	X-linked inhibitor of apoptosis protein
NSA2	Nop seven-associated 2
EGFR	Epidermal growth factor receptor
KRAS	Kirsten rat sarcoma viral oncogene homolog
SRB	Sulforhodamine B
MTT	3-(4,5)-dimethylthiahiazo (-z-y1)-3,5-di- phenytetrazoliumromide
PARP	Poly ADP-ribose polymerase
Bax	Bcl-2-associated X protein
Stat3	Signal transducer and activator of transcription 3
AKT	Protein Kinase B
ERK	Extracellular-regulated protein kinase

## References

[1] Siegel R.L., Miller K.D., Jemal A. (2018). Cancer statistics 2018. CA Cancer J. Clin..

[2] Schrank Z., Chhabra G., Lin L., Iderzorig T., Osude C., Khan N., Kuckovic A., Singh S., Miller R.J., Puri N. (2018). Current Molecular-Targeted Therapies in NSCLC and Their Mechanism of Resistance. Cancers.

[3] Favaretto A., Pasello G., Magro C., Schettino C., Gridelli C. (2009). Second and third line treatment in non-small cell lung cancer. Crit. Rev. Oncol. Hematol..

[4] Chen Z., Fillmore C.M., Hammerman P.S., Kim C.F., Wong K.K. (2014). Non-small-cell lung cancers: A heterogeneous set of diseases. Nat. Rev. Cancer.

[5] Imyanitov E.N., Iyevleva A.G., Levchenko E.V. (2021). Molecular testing and targeted therapy for non-small cell lung cancer: Current status and perspectives. Crit. Rev. Oncol. Hematol..

[6] Imielinski M., Berger A.H., Hammerman P.S., Hernandez B., Pugh T.J., Hodis E., Cho J., Suh J., Capelletti M., Sivachenko A. (2012). Mapping the hallmarks of lung adenocarcinoma with massively parallel sequencing. Cell.

[7] Kadara H., Choi M., Zhang J., Parra E.R., Rodriguez-Canales J., Gaffney S.G., Zhao Z., Behrens C., Fujimoto J., Chow C. (2017). Whole-exome sequencing and immune profiling of early-stage lung adenocarcinoma with fully annotated clinical follow-up. Ann. Oncol..

[8] Bodor J.N., Boumber Y., Borghaei H. (2020). Biomarkers for immune checkpoint inhibition in non-small cell lung cancer (NSCLC). Cancer.

[9] Pommier Y. (2006). Topoisomerase I inhibitors: Camptothecins and beyond. Nat. Rev. Cancer.

[10] Liew S.T., Yang L.X. (2008). Design, synthesis and development of novel camptothecin drugs. Curr. Pharm. Des..

[11] Li Q.Y., Zu Y.G., Shi R.Z., Yao L.P. (2006). Review camptothecin: Current perspectives. Curr. Med. Chem..

[12] Zunino F., Dallavalleb S., Laccabuea D., Berettaa G., Merlinib L., Pratesi G. (2002). Current status and perspectives in the development of camptothecins. Curr. Pharm. Des..

[13] Ling X., Cao S., Cheng Q., Keefe J.T., Rustum Y.M., Li F. (2012). A novel small molecule FL118 that selectively inhibits surviving, Mcl-1, XIAP and cIAP2 in a p53-independent manner, shows superior antitumor activity. PLoS ONE.

[14] Li F. (2014). Anticancer drug FL118 is more than a survivin inhibitor: Where is the Achilles’ heel of cancer?. Am. J. Cancer Res..

[15] Zhao J., Ling X., Cao S., Liu X., Wan S., Jiang T., Li F. (2014). Antitumor activity of FL118, a surviving, Mcl-1, XIAP, and cIAP2 selective inhibitor, is highly dependent on its primary structure and steric configuration. Mol. Pharm..

[16] Ling X., Xu C., Fan C., Zhong K., Li F., Wang X. (2014). FL118 induces p53-dependent senescence in colorectal cancer cells by promoting degradation of MdmX. Cancer Res..

[17] Wu G., Mai X., Liu F., Lin M., Dong X., Xu Q., Hao C., Zhang L., Yu R., Jiang T. (2019). Synthesis of novel 10,11-methylenedioxy-camptothecin glycoside derivatives and investigation of their anti-tumor effects in vivo. RSC Adv..

[18] Pommier Y., Kohlhagen G., Kohn K.W., Leteurtre F., Wani M.C., Wall M.E. (1995). Interaction of an alkylating camptothecin derivative with a DNA base at topoisomerase I-DNA cleavage sites. Proc. Natl. Acad. Sci. USA.

[19] Dallavalle S., Delsoldato T., Ferrari A., Merlini L., Penco S., Carenini N., Perego P., De Cesare M., Pratesi G., Zunino F. (2000). Novel 7-substituted camptothecins with potent antitumor activity. J. Med. Chem..

[20] Dallavalle S., Merlini L., Morini G., Musso L., Penco S., Beretta G.L., Tinelli S., Zunino F. (2004). Synthesis and cytotoxic activity of substituted 7-aryliminomethyl derivatives of camptothecin. Eur. J. Med. Chem..

[21] Perego P., De Cesare M., De Isabella P., Carenini N., Beggiolin G., Pezzoni G., Palumbo M., Tartaglia L., Pratesi G., Pisano C. (2001). A novel 7-modified camptothecin analog overcomes breast cancer resistance protein-associated resistance in a mitoxantrone-selected colon carcinoma cell line. Cancer Res..

[22] De Cesare M., Beretta G.L., Tinelli S., Benedetti V., Pratesi G., Penco S., Dallavalle S., Merlini L., Pisano C., Carminati P. (2007). Preclinical efficacy of ST1976, a novel camptothecin analog of the 7-oxyiminomethyl series. Biochem. Pharmacol..

[23] Pisano C., De Cesare M., Beretta G.L., Zuco V., Pratesi G., Penco S., Vesci L., Fodera R., Ferrara F.F., Guglielmi M.B. (2008). Preclinical profile of antitumor activity of a novel hydrophilic camptothecin, ST1968. Mol. Cancer Ther..

[24] Zhang G., Yin R., Dai X., Wu G., Qi X., Yu R., Li J., Jiang T. (2022). Design, synthesis, and biological evaluation of novel 7-substituted 10,11-methylenedioxy-camptothecin derivatives against drug-resistant small-cell lung cancer in vitro and in vivo. Eur. J. Med. Chem..

[25] Ling X., Liu X., Zhong K., Smith N., Prey J., Li F. (2015). FL118, a novel camptothecin analogue, overcomes irinotecan and topotecan resistance in human tumor xenograft models. Am. J. Transl. Res..

[26] Boulares A.H., Yakovlev A.G., Ivanova V., Stoica B.A., Wang G., Iyer S., Smulson M. (1999). Role of poly(ADP-ribose) polymerase (PARP) cleavage in apoptosis. Caspase 3-resistant PARP mutant increases rates of apoptosis in transfected cells. J. Biol. Chem..

[27] Boice A., Bouchier-Hayes L. (2020). Targeting apoptotic caspases in cancer. Biochim. Biophys. Acta Mol. Cell Res..

[28] Cosentino K., Hertlein V., Jenner A., Dellmann T., Gojkovic M., Pena-Blanco A., Dadsena S., Wajngarten N., Danial J.S.H., Thevathasan J.V. (2022). The interplay between BAX and BAK tunes apoptotic pore growth to control mitochondrial-DNA-mediated inflammation. Mol. Cell.

[29] Song S., Jacobson K.N., McDermott K.M., Reddy S.P., Cress A.E., Tang H., Dudek S.M., Black S.M., Garcia J.G., Makino A. (2016). ATP promotes cell survival via regulation of cytosolic [Ca^2+^] and Bcl-2/Bax ratio in lung cancer cells. Am. J. Physiol. Cell Physiol..

[30] Yu J.W., Shi Y. (2008). FLIP and the death effector domain family. Oncogene.

[31] Liu L.F., Desai S.D., Li T.K., Mao Y., Sun M., Sim S.P. (2000). Mechanism of action of camptothecin. Ann. N. Y. Acad. Sci..

[32] Li J., Huang H.Y., Lin Y.C., Zuo H., Tang Y., Huang H.D. (2023). *Cinnamomi ramulus* inhibits cancer cells growth by inducing G2/M arrest. Front. Pharmacol..

[33] Huang Y.G., Li D., Wang L., Su X.M., Tang X.B. (2022). CENPF/CDK1 signaling pathway enhances the progression of adrenocortical carcinoma by regulating the G2/M-phase cell cycle. J. Transl. Med..

[34] Mohassab A.M., Hassan H.A., Abdelhamid D., Abdel-Aziz M. (2020). STAT3 transcription factor as target for anti-cancer therapy. Pharmacol. Rep..

[35] Miricescu D., Totan A., Stanescu S., Badoiu S.C., Stefani C., Greabu M. (2020). PI3K/AKT/mTOR Signaling Pathway in Breast Cancer: From Molecular Landscape to Clinical Aspects. Int. J. Mol. Sci..

[36] Sugiura R., Satoh R., Takasaki T. (2021). ERK: A Double-Edged Sword in Cancer. ERK-Dependent Apoptosis as a Potential Therapeutic Strategy for Cancer. Cells.

[37] Wang X.J., Yu J., Wong S.H., Cheng A.S., Chan F.K., Ng S.S., Cho C.H., Sung J.J., Wu W.K. (2013). A novel crosstalk between two major protein degradation systems: Regulation of proteasomal activity by autophagy. Autophagy.

[38] Myung J., Kim K.B., Crews C.M. (2001). The ubiquitin-proteasome pathway and proteasome inhibitors. Med. Res. Rev..

[39] Ding J., Li X., Khan S., Zhang C., Gao F., Sen S., Wasylishen A.R., Zhao Y., Lozano G., Koul D. (2022). EGFR suppresses p53 function by promoting p53 binding to DNA-PKcs: A noncanonical regulatory axis between EGFR and wild-type p53 in glioblastoma. Neuro. Oncol..

[40] Li W., Song A.P., Zhao F., Hu Y.M., Hua M. (2013). A novel human TINP1 gene promotes cell proliferation through inhibition of p53 and p21 expression. Oncol. Rep..

[41] Chan C.H., Chiou L.W., Lee T.Y., Liu Y.R., Hsieh T.H., Yang C.Y., Jeng Y.M. (2023). PAK and PI3K pathway activation confers resistance to KRAS(G12C) inhibitor sotorasib. Br. J. Cancer.

[42] Liu Y.Q., Li W.Q., Morris-Natschke S.L., Qian K., Yang L., Zhu G.X., Wu X.B., Chen A.L., Zhang S.Y., Nan X. (2015). Perspectives on biologically active camptothecin derivatives. Med. Res. Rev..

[43] Ling X., Wu W., Aljahdali I.A.M., Liao J., Santha S., Fountzilas C., Boland P.M., Li F. (2022). FL118, acting as a ‘molecular glue degrader’, binds to dephosphorylates and degrades the oncoprotein DDX5 (p68) to control c-Myc, survivin and mutant Kras against colorectal and pancreatic cancer with high efficacy. Clin. Transl. Med..

[44] Soria J.C., Ohe Y., Vansteenkiste J., Reungwetwattana T., Chewaskulyong B., Lee K.H., Dechaphunkul A., Imamura F., Nogami N., Kurata T. (2018). Osimertinib in Untreated EGFR-Mutated Advanced Non-Small-Cell Lung Cancer. N. Engl. J. Med..

[45] Pore M.M., Hiltermann T.J., Kruyt F.A. (2013). Targeting apoptosis pathways in lung cancer. Cancer Lett..

[46] Steelman L.S., Abrams S.L., Whelan J., Bertrand F.E., Ludwig D.E., Basecke J., Libra M., Stivala F., Milella M., Tafuri A. (2008). Contributions of the Raf/MEK/ERK, PI3K/PTEN/Akt/mTOR and Jak/STAT pathways to leukemia. Leukemia.

[47] Scaltriti M., Baselga J. (2006). The epidermal growth factor receptor pathway: A model for targeted therapy. Clin. Cancer Res..

[48] Ye M., Zhang Y., Gao H., Xu Y., Jing P., Wu J., Zhang X., Xiong J., Dong C., Yao L. (2018). Activation of the Aryl Hydrocarbon Receptor Leads to Resistance to EGFR TKIs in Non-Small Cell Lung Cancer by Activating Src-Mediated Bypass Signaling. Clin. Cancer Res..

[49] Amodio V., Yaeger R., Arcella P., Cancelliere C., Lamba S., Lorenzato A., Arena S., Montone M., Mussolin B., Bian Y. (2020). EGFR Blockade Reverts Resistance to KRAS Inhibition in Colorectal Cancer. Cancer Discov..

[50] Gudkov A.V., Komarova E.A. (2003). The role of p53 in determining sensitivity to radiotherapy. Nat. Rev. Cancer.

[51] Weller M. (1998). Predicting response to cancer chemotherapy: The role of p53. Cell Tissue Res..

[52] Fischer M., Quaas M., Steiner L., Engeland K. (2016). The p53-p21-DREAM-CDE/CHR pathway regulates G2/M cell cycle genes. Nucleic Acids Res..

[53] Taylor W.R., Stark G.R. (2001). Regulation of the G2/M transition by p53. Oncogene.

[54] Wei H., Qu L., Dai S., Li Y., Wang H., Feng Y., Chen X., Jiang L., Guo M., Li J. (2021). Structural insight into the molecular mechanism of p53-mediated mitochondrial apoptosis. Nat. Commun..

[55] Viktorsson K., De Petris L., Lewensohn R. (2005). The role of p53 in treatment responses of lung cancer. Biochem. Biophys. Res. Commun..

[56] Mogi A., Kuwano H. (2011). TP53 mutations in nonsmall cell lung cancer. BioMed Res. Int..

[57] Ahrendt S.A., Hu Y., Buta M., McDermott M.P., Benoit N., Yang S.C., Wu L., Sidransky D. (2003). p53 mutations and survival in stage I non-small-cell lung cancer: Results of a prospective study. J. Natl. Cancer Inst..

[58] Zhang H., Ma X., Shi T., Song Q., Zhao H., Ma D. (2010). NSA2, a novel nucleolus protein regulates cell proliferation and cell cycle. Biochem. Biophys. Res. Commun..

