# Supplementary material for: Novel Camptothecin Derivative 9c with Enhanced Antitumor Activity via NSA2-EGFR-P53 Signaling Pathway
Source: Int J Mol Sci. 2025 Feb 25;26(5):1987. doi: 10.3390/ijms26051987 (PMC11900506; doi:10.3390/ijms26051987)
Supplement: Supplementary file 1 [file ijms-26-01987-s001.zip › ijms-3456239-supplementary.pdf]

Novel Camptothecin Derivative 9c with Enhanced Antitumor Activity via NSA2-EGFR-P53 Signaling Pathway

Fu Du [1,2,†], Aotong Zhang [1,2,†], Xin Qi [1,2], Ruijuan Yin [1,2], Tao Jiang [1,2,*] and Jing Li [1,2,*]

1 Key Laboratory of Marine Drugs, Chinese Ministry of Education, School of Medicine and Pharmacy, Ocean University of China, Qingdao 266003, China; dufu@stu.ouc.edu.cn (F.D.); zhangaotong6069@stu.ouc.edu.cn (A.Z.); qixin_ouc@ouc.edu.cn (X.Q.); yrj928@163.com (R.Y.)

2 Laboratory for Marine Drugs and Bioproducts of Qingdao National, Laboratory for Marine Science and Technology, Qingdao 266003, China

* Correspondence: jiangtao@ouc.edu.cn (T.J.); lijing_ouc@ouc.edu.cn (J.L.); Tel.: +86-0532-8203-1980 (J.L.)

† These authors contributed equally to this work.

Figure S1: 9c inhibits cell proliferation by blocking cell cycle at the G2/M phase in H1975 cells.
Figure S2: 9c promotes apoptosis of H1975 cells.
Figure S3: 9c inhibits the EGFR- PI3K-AKT pathway in H1975 cells
Figure S4: 9c destabilizes NSA2-EGFR axis in P53 mutant cells.

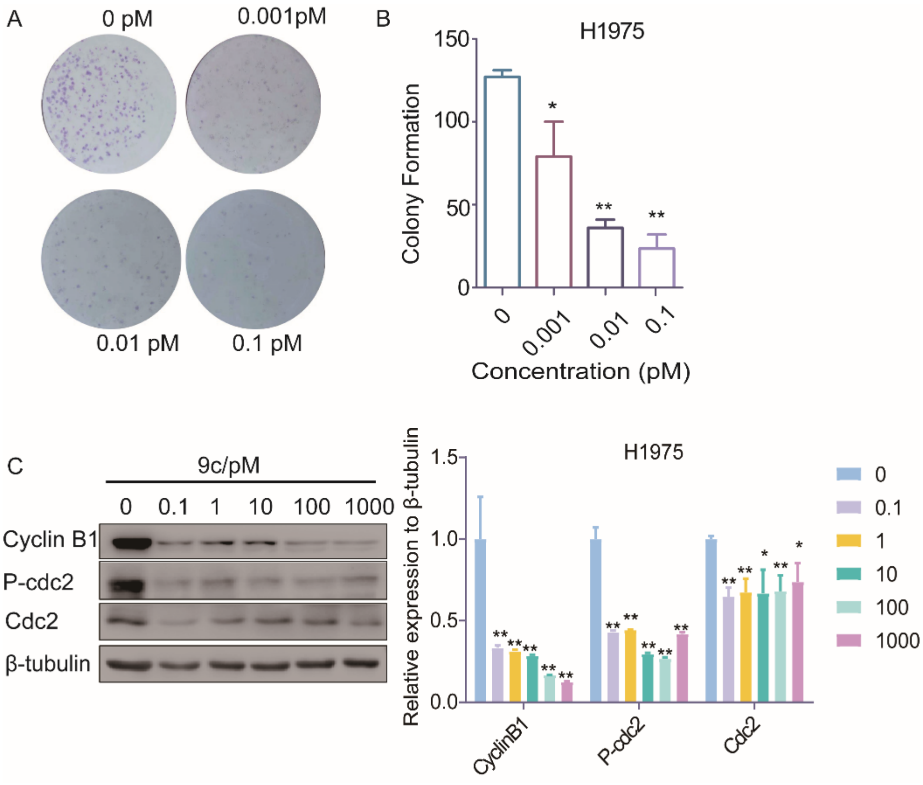

Supplementary Figure S1. 9c inhibits cell proliferation by blocking cell cycle at the G2/M phase in H1975 cells. (A-B) **9c** inhibits the colony formation. Cells were treated with **9c** (0.001–0.1pM) for 14 days, and stained with Giemsa. Then colonies were photographed and counted. One

well plate scale=35 mm. (B)Quantification of the number of colonies. Colonies were scored. Data are presented as means ± SD from three independent experiments. * $p < 0.05$, ** $p < 0.01$. **9c** vs 0 pM group. (C) Compound **9c** inhibited proteins related to G2/M phase cells. The expression of cell cycle-related proteins after treatment with **9c** was determined by Western blot assay; β-tubulin was chosen as the internal control. Protein band densities were quantified by normalizing to β-tubulin (right panel). * $p < 0.05$, ** $p < 0.01$. **9c** vs 0 pM group.

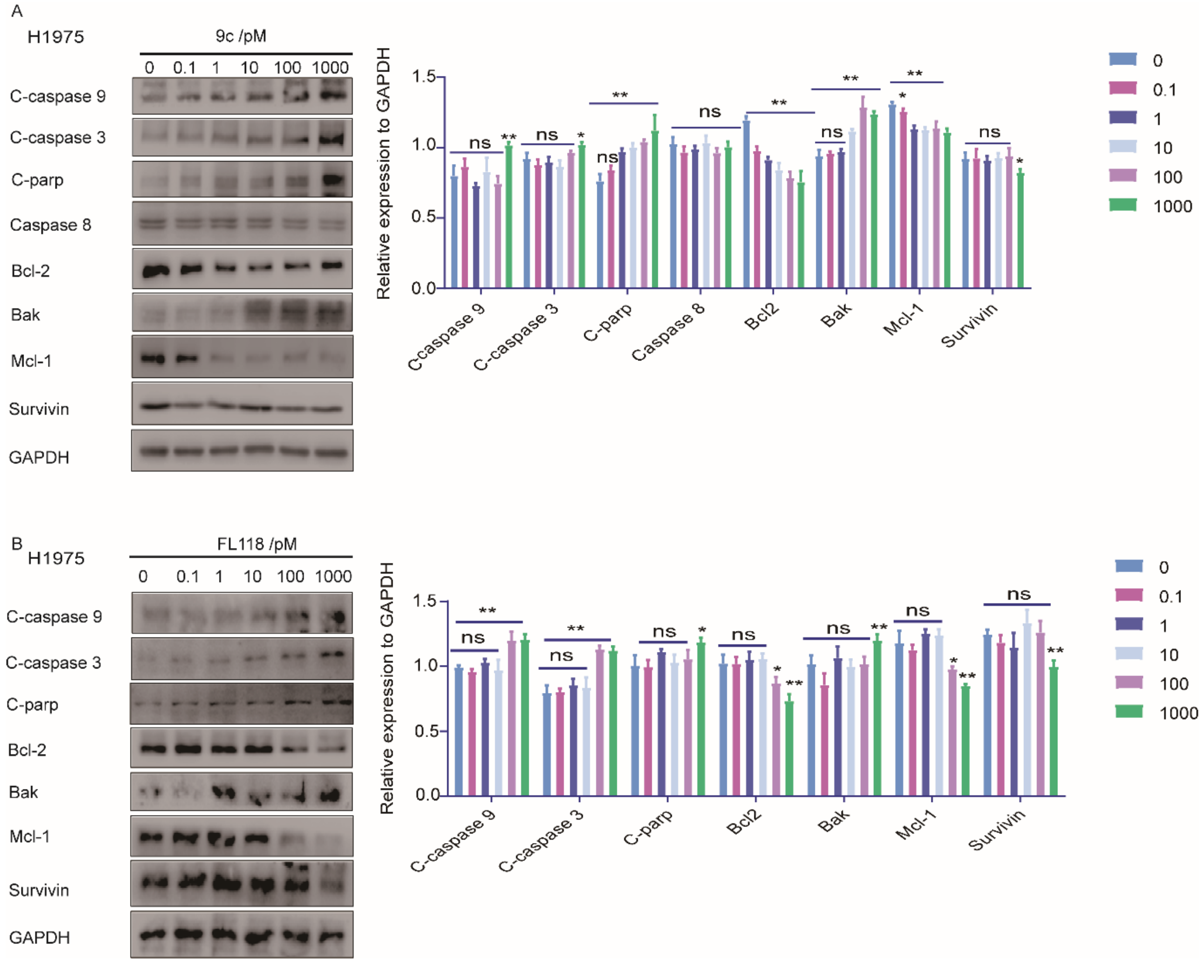

Supplementary Figure S2. 9c promotes apoptosis of H1975 cells. (A-B) Western blot analysis of the expression level of apoptosis-related proteins in indicated cells after treated with **9c** or **FL118** for 24h. GAPDH was chosen as the internal control. Protein band densities were quantified by normalizing to GAPDH (right panel). ns, not statistically significant $p>0.05$,* $p < 0.05$, ** $p < 0.01$,**9c** vs 0 pM group.

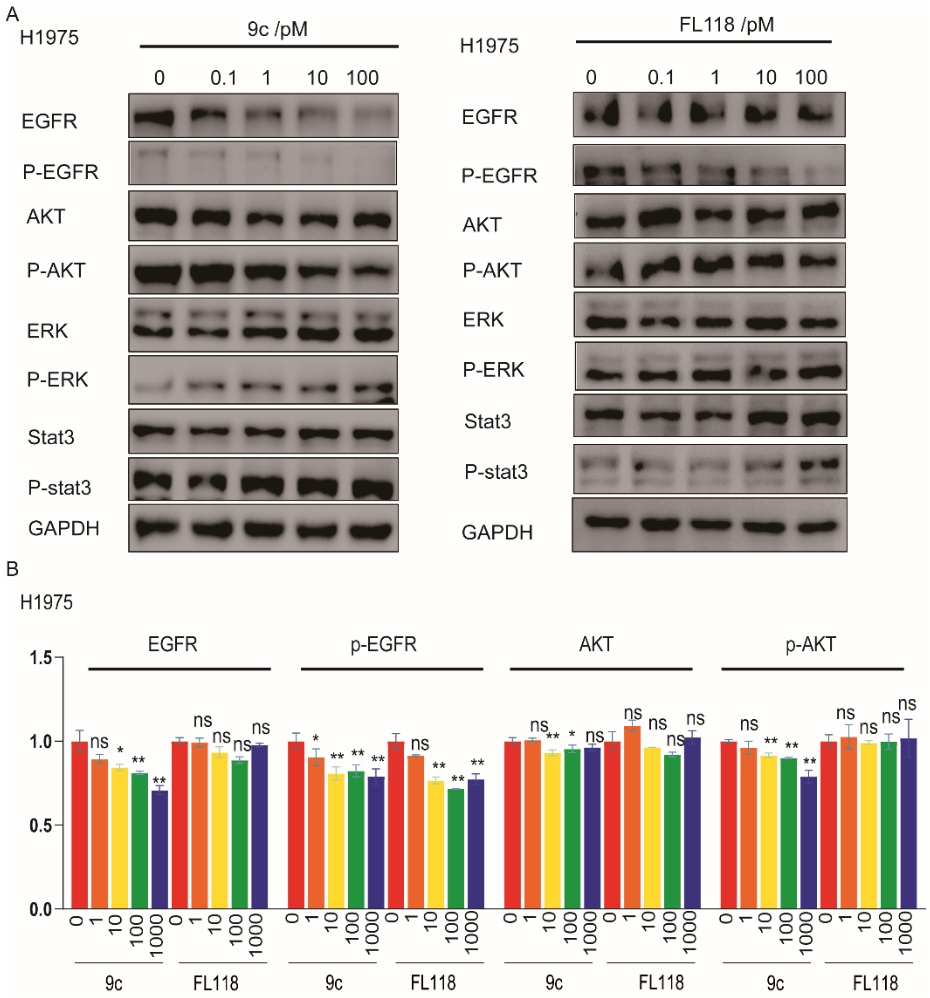

Supplementary Figure S3. 9c inhibits the EGFR- PI3K-AKT pathway. (A) Effects of **9c** on the expressions of signaling molecules related to proliferation. H1975 cells were treated with **9c** (A) or **FL118** (B) for 24 h. GAPDH was used as a loading control. (B) Protein band densities were quantified by normalizing to GAPDH. ns, not statistically significant p>0.05, * p < 0.05, ** p < 0.01 ,**9c** vs 0 pM group.

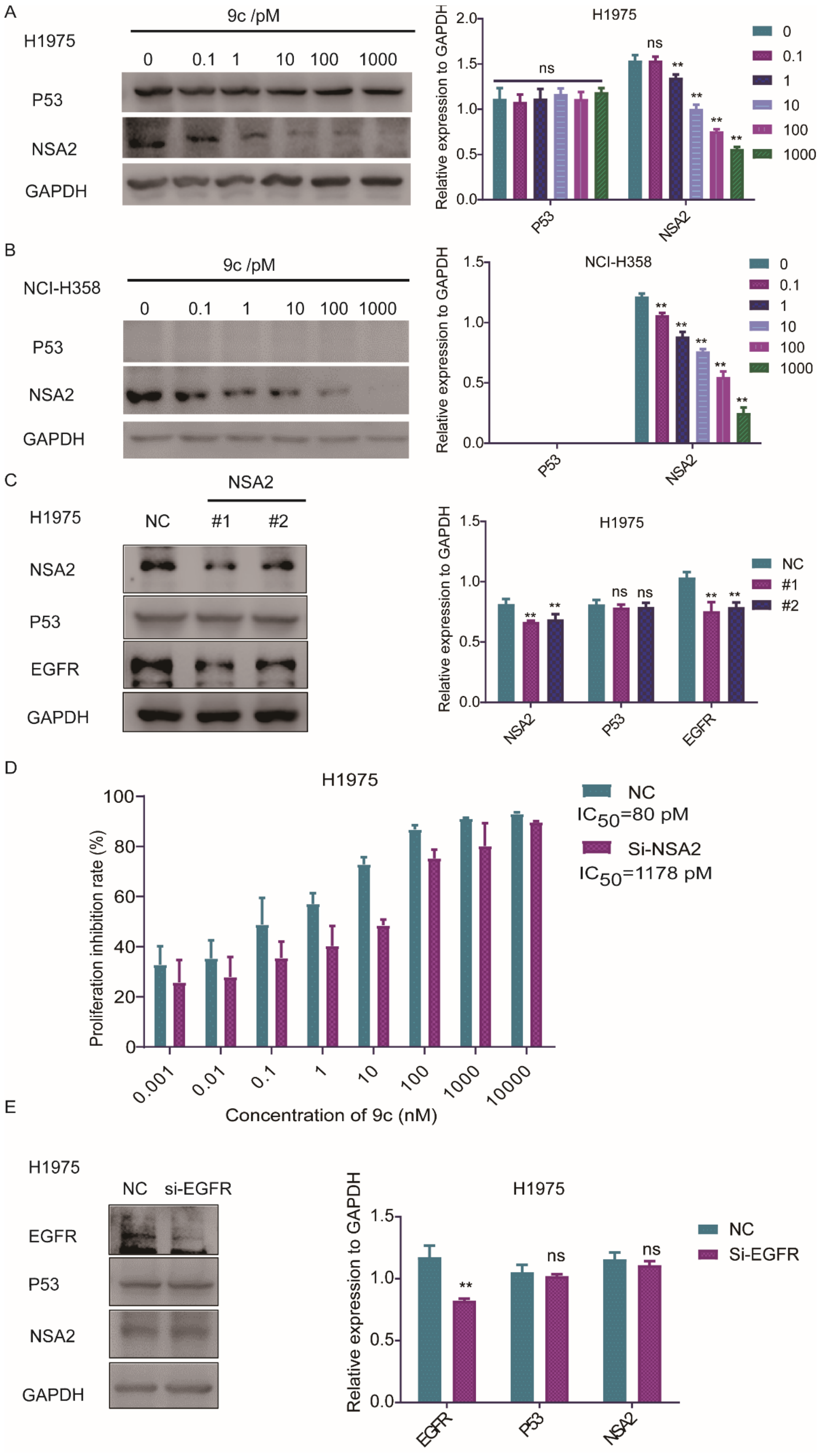
A
H1975
9c /pM
0 0.1 1 10 100 1000
P53
NSA2
GAPDH
H1975
Relative expression to GAPDH
2.0
1.5
1.0
0.5
0.0
ns
ns
**
P53
NSA2
0
0.1
1
10
100
1000
B
NCI-H358
9c /pM
0 0.1 1 10 100 1000
P53
NSA2
GAPDH
NCI-H358
Relative expression to GAPDH
1.5
1.0
0.5
0.0
**
P53
NSA2
C
H1975
NSA2
NC #1 #2
NSA2
P53
EGFR
GAPDH
H1975
Relative expression to GAPDH
1.5
1.0
0.5
0.0
**
ns
NSA2
P53
EGFR
NC
#1
#2
D
H1975
Proliferation inhibition rate (%)
100
80
60
40
20
0
0.001
0.01
0.1
1
10
100
1000
10000
Concentration of 9c (nM)
NC
IC50=80 pM
Si-NSA2
IC50=1178 pM
E
H1975
NC si-EGFR
EGFR
P53
NSA2
GAPDH
H1975
Relative expression to GAPDH
1.5
1.0
0.5
0.0
**
ns
EGFR
P53
NSA2
NC
Si-EGFR

Supplementary Figure S4. **9c** destabilizes NSA2-EGFR axis in P53 mutant cells. (A-B) Effects of **9c** on the expressions of p53 and NSA2. H1975 and H358 cells were treated with **9c** for indicated time. GAPDH was used as a loading control. ns, not statistically significant $p>0.05$, **p < 0.01.**9c** vs 0 pM group. (C) H1975 cells were transfected with the NSA2 targeting siRNA using Lipo3000 transfection reagents. After 48 h, the protein levels were measured by Western blot analysis. GAPDH was used as a loading control. Protein band densities were quantified by normalizing to GAPDH (right panel). ns, not statistically significant $p>0.05$, ** p < 0.01.NC vs si-NSA2 .(D) H1975 cells were transfected with the NSA2 targeting siRNA using Lipo3000 transfection reagents. After 48 h, the transfection efficiency was examined by Western blot. H1975 cells with NSA2-knockdown were treated with indicated concentrations of **9c** for 72 h and cell viability was determined by SRB Assay. (E)H1975 cells were transfected with the EGFR targeting siRNA using Lipo3000 transfection reagents. After 48 h, the protein levels were measured by Western blot analysis. GAPDH was used as a loading control. Protein band densities were quantified by normalizing to GAPDH (right panel).ns, not statistically significant $p>0.05$, ** p < 0.01.NC vs si-EGFR.
